# Structure-based learning to predict and model protein–DNA interactions and transcription-factor co-operativity in *cis*-regulatory elements

**DOI:** 10.1093/nargab/lqae068

**Published:** 2024-06-12

**Authors:** Oriol Fornes, Alberto Meseguer, Joachim Aguirre-Plans, Patrick Gohl, Patricia M Bota, Ruben Molina-Fernández, Jaume Bonet, Altair Chinchilla-Hernandez, Ferran Pegenaute, Oriol Gallego, Narcis Fernandez-Fuentes, Baldo Oliva

**Affiliations:** Centre for Molecular Medicine and Therapeutics. BC Children's Hospital Research Institute. Department of Medical Genetics. University of British Columbia, Vancouver, BC V5Z 4H4, Canada; Structural Bioinformatics Lab (GRIB-IMIM). Department of Medicine and Life Sciences, Universitat Pompeu Fabra, Barcelona 08005 Catalonia, Spain; Center for Complex Network Research. Northeastern University, Boston, MA 02115, USA; Structural Bioinformatics Lab (GRIB-IMIM). Department of Medicine and Life Sciences, Universitat Pompeu Fabra, Barcelona 08005 Catalonia, Spain; Structural Bioinformatics Lab (GRIB-IMIM). Department of Medicine and Life Sciences, Universitat Pompeu Fabra, Barcelona 08005 Catalonia, Spain; Structural Bioinformatics Lab (GRIB-IMIM). Department of Medicine and Life Sciences, Universitat Pompeu Fabra, Barcelona 08005 Catalonia, Spain; Structural Bioinformatics Lab (GRIB-IMIM). Department of Medicine and Life Sciences, Universitat Pompeu Fabra, Barcelona 08005 Catalonia, Spain; Laboratory of Protein Design & Immunoengineering. School of Engineering. Ecole Polytechnique Federale de Lausanne. Lausanne 1015, Vaud, Switzerland; Live-Cell Structural Biology. Department of Medicine and Life Sciences, Universitat Pompeu Fabra, Barcelona 08005 Catalonia, Spain; Live-Cell Structural Biology. Department of Medicine and Life Sciences, Universitat Pompeu Fabra, Barcelona 08005 Catalonia, Spain; Live-Cell Structural Biology. Department of Medicine and Life Sciences, Universitat Pompeu Fabra, Barcelona 08005 Catalonia, Spain; Institute of Biological, Environmental and Rural Science. Aberystwyth University, SY23 3DA Aberystwyth, UK; Structural Bioinformatics Lab (GRIB-IMIM). Department of Medicine and Life Sciences, Universitat Pompeu Fabra, Barcelona 08005 Catalonia, Spain

## Abstract

Transcription factor (TF) binding is a key component of genomic regulation. There are numerous high-throughput experimental methods to characterize TF–DNA binding specificities. Their application, however, is both laborious and expensive, which makes profiling all TFs challenging. For instance, the binding preferences of ∼25% human TFs remain unknown; they neither have been determined experimentally nor inferred computationally. We introduce a structure-based learning approach to predict the binding preferences of TFs and the automated modelling of TF regulatory complexes. We show the advantage of using our approach over the classical nearest-neighbor prediction in the limits of remote homology. Starting from a TF sequence or structure, we predict binding preferences in the form of motifs that are then used to scan a DNA sequence for occurrences. The best matches are either profiled with a binding score or collected for their subsequent modeling into a higher-order regulatory complex with DNA. Co-operativity is modelled by: (i) the co-localization of TFs and (ii) the structural modeling of protein–protein interactions between TFs and with co-factors. We have applied our approach to automatically model the interferon-β enhanceosome and the pioneering complexes of OCT4, SOX2 (or SOX11) and KLF4 with a nucleosome, which are compared with the experimentally known structures.

## Introduction

Transcriptional regulatory elements are key players of the genome during development, cell and tissue homeostasis, responses to external stimuli, and disease (1). Unravelling the mechanisms that regulate gene expression has consequently become one of the major challenges in Biology. With this objective the increase in the scale of experimental data, across multiple data types, has provided a plethora of activating regulatory elements of the genome (2). Classical definitions of activating regulatory elements are focused in two classes: promoters (where transcription is initiated) and enhancers (elements that amplify such transcription initiation in *cis*, i.e. located within less than 1M bases distance of the initiation). However, this distinction is becoming increasingly unclear, suggesting an updated model based on DNA accessibility of binding sites and enhancer/promoter potential (1). The sequence preferences of transcription factors (TFs) for these binding sites can be assessed by a wide variety of experimental techniques, both *in vitro* (such as SELEX (3,4), SMiLE-SEQ (5), protein-binding microarrays (PBM) (6–8) and MPRA (9,10)) and *in vivo* (such as bacterial and yeast one hybrid assays (11,12), ChIP-Seq (13) and other high-throughput techniques (14,15)). Recent models show similar potential of enhancers and promoters to promote the transcription machinery. Andersson et al. (1) have pointed towards the TF and RNA polymerase II-centric cooperative model, in which regulatory elements work together to increase or maintain the local concentrations of transcription factors (TFs), RNA polymerase II (RNAPII), and other co-factors, thereby increasing the probability to target gene transcription start sites. Besides, it appears that very few proteins in humans occupy most of their motif matches under physiological conditions (16), which highlights the importance of the balance between the co-operativity of TFs and their strength upon binding. Co-operative recognition of DNA by multiple TFs defines unique genomic positions on the genome and confers a systemic stability of regulation. Co-operative binding is most easily understood when it is mediated by protein–protein interactions that confer additional stability when two (or more) interacting proteins bind DNA (16). Most eukaryotic TFs recruit cofactors as ‘coactivators’ or ‘corepressors’ forming large protein complexes to regulate transcription (17). They commonly contain domains involved in chromatin binding, nucleosome remodelling and/or covalent modification of histones or other proteins (16). In the absence of direct protein–protein contacts between TFs, co-operativity can be mediated through DNA. Using CAP-SELEX (18) Jolma *et al.* (19) unveiled *in vitro* the co-operation of pairs of TFs through protein–protein and protein–DNA interactions. However, experimental protocols are both laborious and difficult to apply, and consequently most high-throughput efforts have been focused on a limited number of organisms.

Here, we have developed a structure-based learning approach to predict TF binding features and model the regulatory complex(es) in *cis-*regulatory modules (i.e. enhancers and promoters). Our objective is to characterize the role of structural elements, taking advantage of the recent developments on protein structure prediction (i.e. AlphaFold (20)) to reinforce both its modelling and prediction. Our approach integrates the experimental knowledge of structures of TF–DNA complexes and the large amount of high-throughput TF–DNA interactions to develop statistical knowledge-based potentials with which to score the binding capability of TFs in *cis-*regulatory elements. We have developed a server to characterize and model the binding specificity of a TF sequence or its structure. The server can automatically produce structural models of TF–DNA interactions and their complexes with co-factors. The approach is applied to the examples of interferon-β enhanceosome (21) and the recent complex of ‘pioneer factors’ SOX11/SOX2 and OCT4 with the nucleosome (22). The model of interferon-β enhanceosome highlights the co-operativity of TFs with a more holistic view of domain-domain interactions that were missed in the experimental structures. The model of the pioneering regulatory complex locates OCT4, which is missing in the experimental structure, suggesting a potential role for nucleosome opening.

## Materials and methods

### Software

We use DSSP (version CMBI 2006) (23) to obtain the secondary structure and surface accessibility; X3DNA (version 2.0) (24) to obtain DNA structures; *matcher* and *needle*, from the EMBOSS package (version 6.6.0) (25), to obtain local and global alignments, respectively; BLAST (version 2.2.31+) (26) to obtain potential homologs of a protein sequence; MODELLER (version 9.9) (27) to model the structure of a protein by homology; TM-align to compare similar TF folds (28); CD-HIT to obtain a non-redundant set of sequences of TFs (29) and the programs FIMO (30) and TOMTOM of the MEME suite (31) to obtain the fragments of a DNA sequence that aligns with a Position-Weight Matrix (PWM) (32) and to compare two PWMs, respectively. The scripts of ModCRE can be downloaded from http://github.com/structuralbioinformatics/ModCRE.

### Databases

Atomic coordinates of protein complexes are retrieved from the PDB repository (33) and protein codes and sequences are extracted from UniProt (34). We only selected the structures of PDB corresponding to TF–DNA interactions. Binding information of TFs was obtained from protein binding microarrays (PBMs) experiments in the Cis-BP database (version 2.00) (35,36). PBMs experiments indicate the binding affinity between TFs and DNA 8-mers with the *E*-score value (between −0.50 and 0.50). DNA 8-mers of a TF with *E*-scores above 0.45 correspond to high affinity interactions (also named positive), while DNA 8-mers with *E*-scores below 0.37 are considered non-bound (or negative); the rest of E-scores are discarded for statistic analyses. For the analyses we use the experimentally known motifs of JASPAR dataset (37).

### Interface of protein–DNA structures

We defined the contacts between TF and DNA using three residues: one amino acid and two contiguous nucleotides of the same strand. The distance of a contact is the distance between the Cβ atom of the amino acid residue and the average position of the atoms of the nitrogen-bases of the two nucleotides and their complementary pairs in the opposite strand (38). Additional features are considered for a contact, such as the secondary structure and solvent accessibility of the amino-acid or the DNA closest groove (major or minor) of the two nucleotides.

### Extending the number of contacts between amino-acids and nucleotides

We used positive DNA 8-mers from PBMs to extend the number of contacts. Briefly, we modelled the interactions of the DNA 8-mers with the TFs using the available structures of TF–DNA pairs in PDB or those of their closest homologs (see details in Supplementary Appendix). We used the TFs of CIS-BP studied by PBM with a known structure in PDB in complex with DNA. We collected the 8-mers with positive interaction (scores higher than 0.45) for each TF, aligned the 8-mers with the DNA sequence of the complex using the PWM of the TF and filtered out the alignments with gaps. We used the alignment to collect new contacts between amino-acids and nucleotides and store them. Then, we checked all other TFs studied by PBM that had a close homolog with known structure in complex with DNA. We used the contacts of the known structures stored above and extended them to the sequences of the homologs using the correspondence of amino-acids obtained from the alignment between protein-sequences. Protein homologs with gaps in the sequence aligned in the region of the interface were removed.

### Contact abundance score

We defined the contact abundance score to capture the increase of coverage in the number and diversity of contacts thanks to the use of PBM experiments. This is defined as the logarithm of the ratio between the total number of potential accessible contacts and the number of contacts at less than 30 Å. For the interaction between an amino-acid and two nucleotides there are 48 different types of contacts (considering the combination of all features, see Supplementary Appendix), therefore the total number of potential contacts is 15 360 (i.e. 42 × 20 × 48), while the number of real contacts depends on the number occurrences in the known structures of a TF family and in the PBMs experiments that can be used to increase them.

### Statistical potentials

We used the definition of statistical potentials described by Feliu *et al.* (39) and Fornes *et al.* (38) applied on the selected database of structures of TF–DNA interactions. These were calculated with the distribution of contacts at <30 Å, using an interval criterion or a distance threshold. We extended the contacts with the experiments of PBMs (see schema of the approach in Figure 9). The interval criterion uses contacts at distances within specific intervals, while the distance threshold accumulates all contacts at distances lower than the threshold. We transformed the statistical potentials into *Z*-scores to identify the contacts with best scores (best distance and best contact residues: i.e. the amino acid and the two nucleotides). The *Z*-score of a contact between one amino-acid and a dinucleotide is obtained by comparison of the score of this contact with the average of scores of all amino-acid substitutions in the same contact and normalized by the standard deviation (see Appendix for details). To avoid redundancies in the statistical potentials we used a criterion of around 70–80% identical contacts for family-specific potentials (i.e. calculated with members of the same TF family sharing the same fold structure) and 40–50% for a general scenario.

### Structural modeling of TF–DNA complexes

Several structural models of a TF–DNA complex were obtained using all its available templates from PDB. First we used BLAST to find the homologs with known structure (template), then the sequence of the query was aligned with the sequences of the templates using MATCHER from the EMBOSS package (25) and a model was built with each template using MODELLER (27). The modeling of the DNA was obtained with the X3DNA package (24) preserving the DNA conformation from the template. This approach required that all the templates used for TF–DNA modeling contained both a TF and a double stranded DNA molecule.

### Construction of PWMs using TF–DNA structural models

We used the *Z*-scores of statistical potentials to obtain the PWM. We selected for each TF family the features optimizing the PWM prediction (see further) and we used the *Z*-score of *ES3DC_dd_* (*ZES3DC_dd_*) as defined in Meseguer *et al.* (40). First, we obtained several models of a TF–DNA interaction using all possible templates. Second, for each model we scored with *ZES3DC_dd_* all the potential DNA sequences of the binding site (i.e. 4^*N*^ sequences, with *N* the size of the binding site, or an alternative heuristic approach as explained in the Supplementary Appendix). Third, we normalized the scores between 0 and 1 and we ranked the DNA sequences. Finally, for each model we selected the sequences with the top scores (this cut-off threshold was also optimized, taking values between 0.7 and 1 in intervals of 0.01). We used the alignment of these sequences to calculate a predicted PWM for each model.

### Optimization of parameters to predict PWMs by grid search

The parameters to predict PWMs that needed to be optimized for each TF family are: (i) the definition of distances’ distribution used to calculate statistical potentials: either by interval-bins (i.e. $x - 1 < d \le x$) or a threshold (i.e. $d \le x$); (ii) the use of a theoretical approach to complete the space of contacts (i.e. using a Taylor's polynomial approach, see Supplementary Appendix); (iii) the dataset of structures used to calculate the potentials: using only the contacts from structures of PDB or adding those from experiments of PBMs; (iv) using a general statistical potential calculated with all known TF–DNA structures or a specific potential calculated with the structures of the same family and fold; (v) the maximum distance to include the contacts of an interface (testing distances at 15, 22 and 30 Å) and (vi) the cut-off threshold to select top ranked DNA sequences used to calculate the PWM (see above). The function to be optimized was the accuracy to predict the PWM of each TF family (i.e. maximum accuracy). A predicted PWM was successful if the alignment with the experimental PWM taken from Cis-BP database was significant (this was calculated with TOMTOM). Then, we selected the parameters that maximized the accuracy of the TF family with the following conditions: (i) maximum number of significant good predictions according to TOMTOM score; (ii) best TOMTOM scores when a similar number of significant solutions were achieved and (iii) the lowest value of the threshold, when several similar solutions were obtained. The parameters selected for each TF family are shown in Table 1. For TFs of the C2H2-ZF family we used the parameters and statistical potentials derived from a previous work (40)

**Table 1.** Table with the number of contacts between amino-acids and dinucleotides, calculated with TF–DNA structures (PDB) and added by models with PBMs data

	**Contacts**		**Parameters for statistical potentials**					
**FAMILY**	PDB	PBM	Contacts’ database	Family	Taylor's approach	BINS	Radius (Å)	MSA threshold
GLOBAL	24651376	762340857	pbm	general	Yes	acc	15	0.82
AP2	1194	3284248	pbm	family	No	acc	15	0.88
C2H2 ZF	224004	35467680	NA*	NA*	No	acc	30	0.94
DM	5114	712912	pbm	family	Yes	acc	22	0.92
E2F	6610	816150	pbm	family	Yes	acc	30	0.84
Ets	92384	26654782	pbm	family	No	bins	30	0.72
Forkhead	64852	20463990	pdb	family	Yes	acc	22	0.84
GATA	28524	7818301	pbm	general	No	acc	15	0.92
Homeodomain	183264	432353402	pbm	family	No	acc	22	0.84
IRF	31656	2285198	pbm	family	No	bins	22	0.88
Myb/SANT	80230	11300750	pdb	family	Yes	acc	22	0.8
NAC/NAM	15098	1980110	pdb	family	No	acc	15	0.76
Nuclear receptor	294588	89311996	pbm	family	No	acc	22	0.96
Sox	189228	5088596	pdb	family	Yes	bins	22	0.76
T-box	40904	6916258	pbm	family	Yes	acc	15	0.8
WRKY	952	777282	pbm	family	No	acc	22	0.82
Zinc cluster	43168	2306230	pbm	family	No	acc	15	0.82
bHLH	61656	13400550	pbm	family	No	acc	30	0.96
bZIP	67216	19605124	pdb	family	Yes	acc	30	0.88

The features selected by grid search for each TF family are shown in the left columns. The row identified as ‘GLOBAL’ shows the total number of contacts with all TFs and the features used for the ‘general’ potential. The table only shows those families with at least 10 different TFs (for all other families see Table S1). *Note: for the C2H2-ZF family we use the statistical potential obtained in a previous work (40).

### Statistical tests of the potentials

We used the Area Under the Precision-Recall Curve (AUPRC) and Area under the Receiver Operating Characteristic Curve (AUROC) to compare the energy-scores of statistical potentials with the PBM scores by analyzing the capacity to distinguish (predict) positive and negative 8-mers of the PBMs experiments. We analyzed the ZES3DC_dd_ statistical potential of TFs of all families from Cis-BP database with PBMs experiments. The protocol for evaluating scoring potentials' efficacy in distinguishing between positive and negative 8-mers comprised several steps. Initially, random selection of negatives and positives was conducted, repeated 10 times for reliability. Then, two unbalanced ratios (1/100 and 1/500) were used to explore the impact of increasing negatives relative to positives. Two studies for negative selection were performed: one maintaining the same G + C content percentage as positives, while the other was random. A 5-fold cross-validation was applied to train and test potentials, evaluating performances under different conditions and obtaining ROC and PR curves. Subsequently, AUROC and AUPRC were calculated, and average values with standard deviation were computed for each condition to provide a comprehensive assessment. Statistical potentials (general and family-specific) were acquired, ensuring each TF wasn’t tested with its own or closely related potentials. Family-specific potentials were defined per structure and fold, limiting identical contacts to 70%, while for general potentials, TFs had less than 40% identical contacts. The study was restricted to TF families with at least 10 different TFs to ensure robust results. Despite filtering out similar interfaces, a relevant number of TFs in the training set had similar interfaces in the testing set of the family-specific potentials. However, the number of TFs in the test of some families was already too small and we could not use a more stringent cut-off without a dramatic loss of applicability. We compared the scores of statistical potentials obtained only with contacts extracted from PDB and the scores obtained with extended contacts using PBMs experiments. We also compared potentials obtained using contacts of TFs of the same family (family-specific) and using a Taylor polynomial approach to complement the missing contacts in the experimental data.

### Nearest neighbor approach

The nearest neighbor approach consists of using the experimental PWM of the closest homolog of a TF (41). This is, we consider that the PWM of a TF (target) is the same as the PWM of another TF (neighbor), selected from a database of TFs with experimentally known PWMs, that has the highest similarity of the protein sequence. The similarity is calculated with the alignment of the sequences of both TFs (target and neighbor). The alignment and comparison of sequences was obtained using MMseq2 (42).

### Implementation of rCLAMPS comparison with ModCRE

rCLAMPS is a recent structural-learning approach developed to predict the PWMs of transcription factors of the homeodomain and C2H2-Zf families (43). We have downloaded and installed from the original github repository, needing the modification of some parameters for running under the same hardware-conditions as ModCRE (see supplementary material for details). We have used the TFs of the homeodomain and C2H2-Zf families from JASPAR to obtain a ROC curve and compare it with ModCRE. TFs with multiple motifs were excluded from this benchmark to avoid misinterpreted false positives. The list of TFs used in this benchmark is in Supplementary File S2. For each TF sequence, we predicted with ModCRE 100 structural models and PWMs, while only one PWM was predicted with rCLAMPS. The comparison with the whole set of non-redundant JASPAR motifs was obtained with TOMTOM and the logarithm of the p-value was used as threshold to select Positives (P) and Negatives (N) of the prediction. True (T) and False (F) predictions were defined according to the comparison with the experimentally known motif of each TF in the list.

### Score of similarity to compare two PWMs

For the comparison between two PWMs we use the program TOMTOM. The score of similarity is defined as $ - lo{g_{10}}( {Pvalue} )$, where the Pvalue is obtained with TOMTOM and it shows if the alignment of the two PWMs is significant (i.e. Pvalue is the probability that a random motif of the same width as the experimental PWM would have an optimal alignment as good or better than the PWM predicted with the structure of the TF)

### Testing the success of predictions by ranking experimental motifs

We use TOMTOM (44) from the MEME suite (31) to compare the PWM predicted of a target with all the experimental motifs from a dataset (i.e. CisBP), including the motif that corresponds to the target. Then, we rank the experimental motifs according to TOMTOM score (from best to worst). The rank of the actual motif of the target indicates the quality of the prediction (see example in Supplementary Figure S9). We transform the rank to fit between 0 and 100, such as the highest score is achieved for the best rank. This yields a normalized score, which is defined as:


\begin{equation*}normal{\mathrm{\;}}score = 100 \times \frac{{\left( {{\mathrm{M}} - rank + 1} \right)}}{{\mathrm{M}}}\end{equation*}

where M is the total amount of PWMs of the dataset. The normalized score is defined null if the Pvalue of the comparison between the predicted PWM and the actual motif is higher than 0.05 (i.e. non-significant). The test will show as successful predictions those with the highest normalized scores (around 100). The normalized score is then calculated as: $normal{\mathrm{\;}}score = 100 \times ( {2638 - rank + 1} )/2638$; as there are 2268 TFs (and 2638 motifs) in the dataset of CisBP with PBM experiments.

### Rank-enrichment prediction

After comparing all the predicted PWMs of a target with the experimental motifs of a dataset, we rank them by the score of TOMTOM, remove all non-significantly aligned PWMs, and select a limited number of motifs from the dataset yielding the top scores. Some motifs in this selected set may have been included several times by their comparison with more than one predicted PWM. Then, we calculate the enrichment of a motif as the ratio of the number of times it appears in the selection. The final prediction (i.e. solution) corresponds to the motif with highest enrichment (i.e. the motif that was more often selected among the predicted PWMs of a target, see example in Supplementary Figure S9). We must note that the choice of the number of motifs selected in the top ranking affects the quality of our prediction, i.e. if we use too many the success is not significant, or in other words, it can be achieved at random (see more details in the Supplementary Appendix).

To evaluate the capacity to predict the correct PWM we use the ranking of the enrichment, transformed into a normalized score between 0 and 100 as above. We calculate the enrichment of the experimental motif of the target and its ranking with respect to the PWM with the highest enrichment (i.e. if the experimental motif of the target has the highest enrichment then the prediction is successful and the normalized score is 100). The normalized score of the experimental motif is the normalized rank-enrichment. If the motif of the target is not selected among the PWMs with the top scores of TOMTOM, then the ranking of the experimental motif cannot be calculated and the prediction is neglected, which reduces the coverage of predictions (i.e. the sensitivity to find solutions). Besides, depending on the number of models used for the enrichment and the total number of acceptable correct solutions (sufficiently similar PWMs), the prediction may not be significant. Thus, non-significant predictions are also neglected and removed from the plots.

### Validation with ChIP-exo experiments

Binding events sufficiently significant (i.e. *P*-value < 0.05) for ChIP-exo experiments from the Gene Transcription Regulation Database (45) (GTRD) were used as a benchmark. Using the ChIP-exo binding start position of the binding site, a ChIP-exo sequence was reconstructed around it (210 bases up and downstream of the binding site) using hg38 (Genome Assembly GRCh38). Each sequence was segmented into 21 bins of 20 nucleotides. The mid bin from position 200–220 contained the true binding site (i.e. this is bin number 11), the surrounding bins each were classified as false bindings. Uniprot Id's from the ChIP experiment were used to retrieve the TF sequence and obtain around 100 structural models. We used ModCRE to predict around 100 PWMs per TF. These Ids were also used to retrieve motifs from JASPAR, using only those with a significant binding by ChIP-exo (they are shown in Supplementary Table S7). For each TF, its PWMs from JASPAR and ModCRE were used to scan the ChIP-exo sequences using FIMO. We calculated the number of PWMs aligning with one or more nucleotides in the bin with FIMO’s P-value lower than a given threshold. Each PWM counted only once in a bin (i.e. if the PWM aligns in two bins only the bin with largest coverage of the PWM is counted). If a bin saw a binding event within, it was labeled as a positive prediction; those bins not witnessing any bases were negative predictions. For ModCRE we only counted as positive the bins with maximum number of aligned PWMs (as there are 100 models, we counted the bin, or bins, with most matches, and the rest were predicted as negative too). When binding was predicted in the middle bin (11 in the above definition) the prediction was true (TP), all other positive predictions were classified as false positives (FP). When no positive prediction occurred within bin 11 that was deemed as false negative (FN), all other negatives were true (TN). We used these definitions to obtain the ROC curves of the selected TFs and their ChIP-exo experiments (codes shown in Table S7) using the binding prediction with JASPAR motifs and models of ModCRE. Then, we averaged the ROC curves of all tests and the standard error was defined as σ/*N*^1/2^, were *N* is the total of tested curves and σ is the standard deviation.

## Results and discussion

### A structure-based learning approach to score TF–DNA interactions

**Figure 1.**
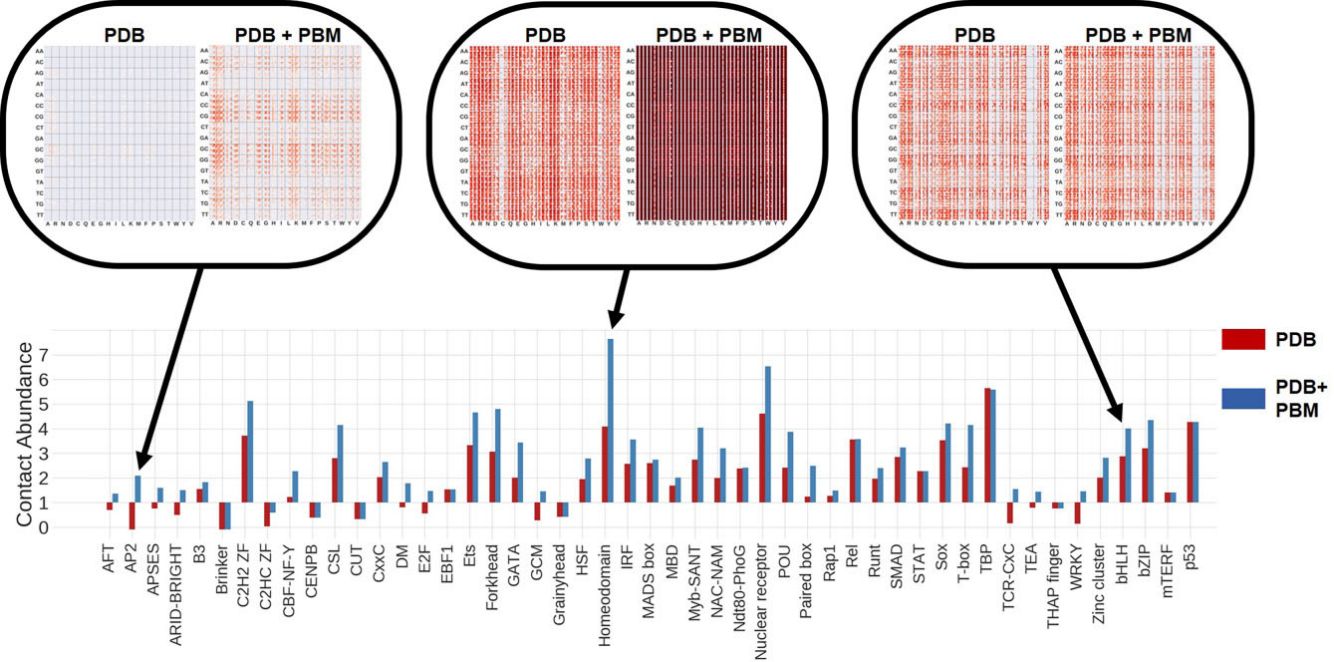
Contact abundance scores of TF families calculated using only the known structures (PDB (60)) or including the potential (modelled) contacts of other TFs derived from PBMs experiments (PDB + PBM). The figure shows in the top the heatmaps of the distribution of contacts for three types of families (AP2, for which the use of PBMs significantly increases the coverage; bHLH, for which the increase is not relevant; and the homeodomain family for which the coverage was already very large with data from PDB). The labels and details of the heatmaps are shown as example in Figure S1. All heatmaps can be downloaded from http://sbi.upf.edu/modcre/##faq.

There are many methods to score the quality of protein folding (46–48) and protein–protein interactions (49,50), such as knowledge-based potentials, also known as statistical potentials (38,51–54). In previous works we developed a set of statistical potentials (55) to analyse protein structures and their interactions (56–58). Ours is a structure-based learning approach that considers the frequency of contacts between pairs of residues and includes their structural environment, such as solvent accessibility and type of secondary structure, to evaluate the interaction between transcription factors (TFs) and nucleic-acids. It is optimized by grid searching to get the best parameters for each TF family. However, a limitation of this approach is the scarcity of known structures and in particular the scarcity of structures of protein–DNA interactions. To overcome this limitation, we developed a method for the C2H2 zinc-fingers (C2H2-ZF) family of TFs (40) that incorporated non-structural experimental information from systematic yeast-one-hybrid (Y1H) experiments (59). Here, we have integrated experimental interactions from protein-binding microarrays (PBMs) for 37 TF families (plus some of their combinations) using the dataset of CisBP (35,36), notably increasing the landscape of protein–DNA contacts shown by the contact-abundance score (see Figure 1). The integration of experimental data from PBMs increased both the number and coverage for different types of contacts over many interval distances. The total number of contacts increased from 24 651 376 contacts to 762 340 857. Table 1 shows the number of contacts obtained with structures from PDB and the increase thanks to PBMs for the most populated TF families (with more than 10 TFs). For example, the use of PBMs data substantially increased the number of contacts for the AP2 and the homeodomain families; however, for the bHLH family the increase was more subtle (see Figure 1 and Table 1). We have named our approach ModCRE (*Mo*delling of *C*is-*R*egulatory *E*lements) and we offer a webservice for its practical use (https://sbi.upf.edu/modcre).

### ModCRE predicts TF binding preferences

We propose two tests to evaluate the predictive power of ModCRE: (i) evaluate the capacity to classify DNA 8-mers as bound/unbound for all TFs of the PBMs experiments and specifically for the TFs of each family and (ii) evaluate the capacity to predict the DNA binding motifs of the TFs in the JASPAR dataset (37) (which are described by means of position weight matrices, i.e. PWM), analyzing the results by TF families. Our objective is to select the binding region of a TF with the purpose of automatically modelling the structure of protein–DNA complexes; therefore, our tests are addressed to check the capacity of recognizing the binding site rather than identifying the critical nucleotides that may be affected by mutations (i.e. characterizing the relevance of each position in the binding site). This goal is achieved by selecting the correct binding or predicting a PWM sufficiently similar to the experimental PWM of a TF target. Further analysis of the binding region and the role of each nucleotide and its position can be addressed in the webserver using the predicted PWMs to scan a DNA sequence uploaded by the user (see in ‘Characterizing/Identifying the binding sites of TFs’).

### Classification of positive 8-mers bindings.

First, we tested the structure-based potential (i.e. named in short *ZES3DC_dd_* in methods and Supplementary Appendix) to discern positive (binding) from negative (non-binding) 8-mers as described in the PBMs experiments of the CisBP database (version 2.0) (36) (see Methods for details). The purpose of this test was to understand the role of the features used to describe the knowledge-based potential. We considered several features, such as the use of Taylor's approach, the use of a general set of contacts collected from structures of the PDB or extended with positive 8-mers from PBMs, or the use of family-specific statistical potentials. We scored the interaction of each TF with all their positive and negative 8-mers by modelling its impact on TF–DNA complexes. We produced precision-recall curves and calculated the area under the curve (AUROC and AUPRC) to compare TF families and the features defining the statistical potential, following the outlined protocols in the methods section. These protocols included testing various unbalanced ratios of negatives to positives and evaluating the influence of G + C content on positive/negative 8-mers. Table 2 displays the average values of AUPRC (Table 2A) and AUROC (Table 2B) with an unbalanced ratio of 1/100 and similar G + C content. Supplementary Tables S2 present the results for 1/100 and 1/500 ratios, with and without similar G + C content. Supplementary Figure S2 displays the distribution of AUPRC and AUROC for all TFs utilizing different potentials. The distributions indicate that statistical potentials calculated specifically for families often yield the best results. However, the Taylor approach only enhances predictions when using contacts from PDB structures, falling short of improving predictions compared to increasing the amount of contacts with experimental data from PBM. Interestingly, some features were better than others depending on the family. Consequently, we designed a grid-search protocol to optimize the best features for each family of TFs to predict their PWMs (see Methods). The best features for each TF family are used as parameters to predict PWMs with ModCRE (see Table 1). They are also calculated for a general statistical potential (with contacts collected from diverse TF families).

**Table 2.** Average values of AUPRC (A) and AUROC (B) in predicting positive/negative 8-mers of the PBMs experiments

*A*								
	General potential				Family specific potential			
	PDB		PDB + PBM		PDB		PDB + PBM	
Family		Taylor		Taylor		Taylor		Taylor
AP2	0.74	0.82	0.75	0.71	0.61	0.81	**0.90**	0.85
C2H2 ZF	0.65	0.66	0.67	0.67	0.70	0.75	0.87	**0.91**
DM	0.71	0.64	0.69	0.50	0.53	0.86	0.87	**0.96**
E2F	0.44	0.41	0.29	0.33	0.84	**0.94**	0.92	0.92
Ets	0.81	0.81	0.88	0.87	0.92	0.93	0.96	**0.98**
Forkhead	0.63	0.63	0.82	0.81	0.68	0.72	**0.90**	**0.89**
GATA	0.55	0.51	0.88	0.84	0.75	0.88	0.92	**0.94**
Homeodomain	0.63	0.62	0.75	0.75	0.77	0.77	**0.91**	0.89
IRF	0.65	0.63	0.83	0.83	0.74	0.89	**0.95**	**0.96**
Myb/SANT	0.65	0.63	0.80	0.80	0.82	0.87	**0.96**	**0.96**
NAC/NAM	0.67	0.68	0.92	0.92	0.67	0.75	**0.96**	0.92
Nuclear receptor	0.69	0.68	0.79	0.79	0.84	0.82	**0.93**	**0.93**
Sox	0.67	0.65	0.77	0.76	0.80	0.80	**0.94**	**0.95**
T-box	0.54	0.51	0.85	0.83	0.78	0.85	**0.97**	0.95
WRKY	0.64	0.64	0.80	0.80	0.83	**0.99**	0.97	**0.98**
Zinc cluster	0.54	0.56	0.62	0.64	0.74	0.88	0.79	**0.92**
bHLH	0.72	0.71	0.90	0.90	0.79	0.84	**0.92**	0.89
bZIP	0.68	0.71	0.79	0.79	0.80	0.78	**0.92**	0.90
***B***								
	**General potential**				**Family specific potential**			
	**PDB**		**PDB + PBM**		**PDB**		**PDB + PBM**	
**Family**		**Taylor**		**Taylor**		**Taylor**		**Taylor**
AP2	0.74	0.82	0.75	0.71	0.61	0.81	**0.90**	0.85
C2H2 ZF	0.65	0.66	0.67	0.67	0.70	0.75	0.87	**0.91**
DM	0.71	0.64	0.69	0.50	0.53	0.86	0.87	**0.96**
E2F	0.44	0.41	0.29	0.33	0.84	**0.94**	0.92	0.92
Ets	0.81	0.81	0.88	0.87	0.92	0.93	0.96	**0.98**
Forkhead	0.63	0.63	0.82	0.81	0.68	0.72	**0.90**	**0.89**
GATA	0.55	0.51	0.88	0.84	0.75	0.88	0.92	**0.94**
Homeodomain	0.63	0.62	0.75	0.75	0.77	0.77	**0.91**	0.89
IRF	0.65	0.63	0.83	0.83	0.74	0.89	**0.95**	**0.96**
Myb/SANT	0.65	0.63	0.80	0.80	0.82	0.87	**0.96**	**0.96**
NAC/NAM	0.67	0.68	0.92	0.92	0.67	0.75	**0.96**	0.92
Nuclear receptor	0.69	0.68	0.79	0.79	0.84	0.82	**0.93**	**0.93**
Sox	0.67	0.65	0.77	0.76	0.80	0.80	**0.94**	**0.95**
T-box	0.54	0.51	0.85	0.83	0.78	0.85	**0.97**	0.95
WRKY	0.64	0.64	0.80	0.80	0.83	**0.99**	0.97	**0.98**
Zinc cluster	0.54	0.56	0.62	0.64	0.74	0.88	0.79	**0.92**
bHLH	0.72	0.71	0.90	0.90	0.79	0.84	**0.92**	0.89
bZIP	0.68	0.71	0.79	0.79	0.80	0.78	**0.92**	0.90

PDB columns display results obtained with potentials derived from contacts in PDB while columns labeled PDB + PBM show results with contacts extended using PBMs experiments. The potentials are categorized as general or family-specific, with or without utilizing Taylor's approach. This study is restricted to those families with at least 10 different TFs to sufficiently support the results, using an unbalanced ratio of 1/100 between positive and negatives with similar G + C. The results of the area under the curve of true and false positive rates (AUROC) are shown in B, using the same column definitions.

### Comparison of PWMs with JASPAR motifs

**Figure 2.**
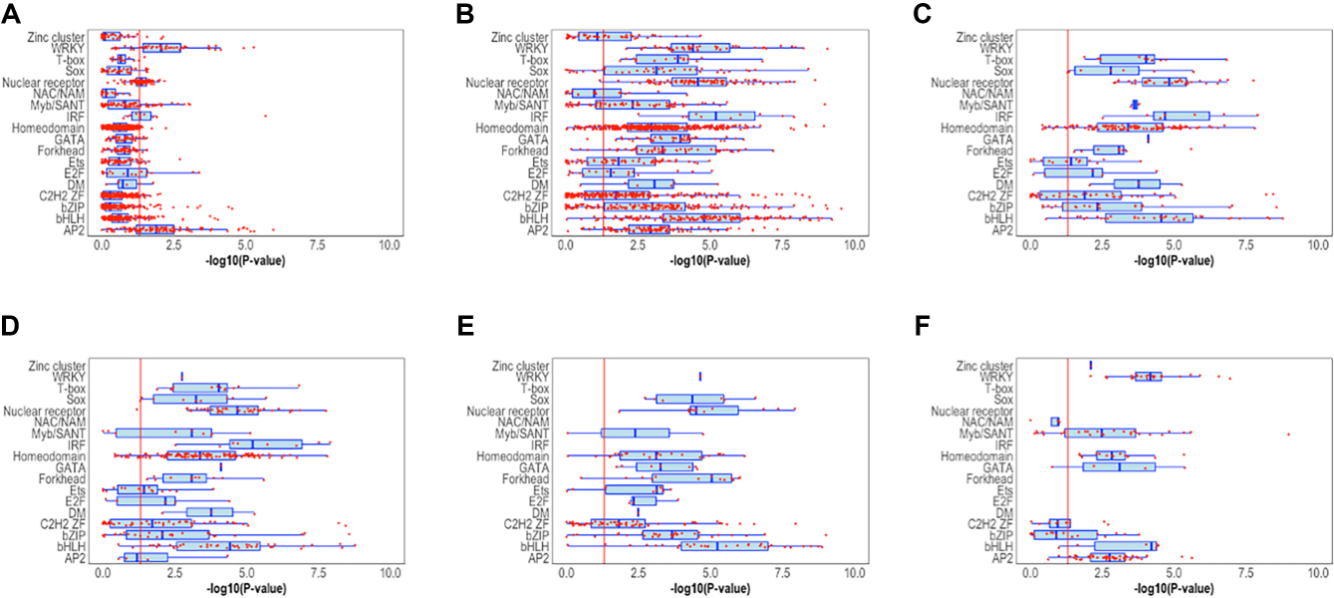
Distribution of ‘similarity scores’ to compare predicted and experimental PWMs of TFs from JASPAR database. A and B show the results for the comparison with any experimental method, C-F shows the result for the comparison with PWMs obtained with specific experiments: HT-SELEX (**C**), SELEX (**D**), ChIP-Seq (**E**) and DAP-Seq (**F**). Each red dot in the plot shows either the average of scores (in A) or the highest score (in B,C,D,E and F) out of 100 predictions. Boxes in blue show the best quartiles of the distribution for each TF, highlighting in red the mean of the distribution for all TFs of a family. A red line indicates the threshold at which the predicted PWM is significantly similar to the experimental (i.e. *P*-value < 0.05). We restricted the study to those families with at least 10 different TF sequences (the comparison with the rest of families of TFs is in Figure S3).

Second, we used these optimized parameters to predict the motifs of TFs in JASPAR dataset (37) which structure could be modelled. Many PWMs in this set were obtained by HT-SELEX, SELEX or other methods such as ChIP-Seq, instead of PBMs. We compared the predicted PWMs with those obtained by experimental methods other than PBMs, thus representing independent testing sets. The structural models of many TFs were obtained with different templates (i.e. using several known structures of close homologs interacting with a double-strand DNA helix). Therefore, to equilibrate the number of models, we limited to 100 the number of models for each TF, obtained by using all available templates from PDB (60) and/or generating several conformations with MODELLER (27). We used the version of JASPAR consisting of 1934 PWMs. We discarded TFs of JASPAR with more than one PWM to avoid misinterpretation of predictions. The final dataset contained 1210 TFs, approximately 60% of the original dataset. We predicted 100 PWMs per TF and compared them with the experimental motif from JASPAR using TOMTOM (31). We obtained the *P-value* provided by TOMTOM from each comparison and transformed it into a measure of similarity (*similarity score*, defined in methods). Figure 2 shows the results of the prediction for several families of TFs by plotting the average (in Figure 2A) and the best scores (in Figure 2B) out of 100 models of each TF. Figure 2C to F show the results with the best scores in the comparison with motifs obtained by HT-SELEX, SELEX, ChIP-seq and DAP-seq. For most TFs (around 80%) ModCRE predicted at least one motif significantly similar to the experimental (*P*-value < 0.05). However, we must note that the criterion of P-value from the comparison of two PWMs may have some intrinsic dependencies (for example on the size of the PWMs or in the variance of DNA binding sequences for different TF families); thus, it is not the best criterion to compare results from different TF families. Besides, we notice that having a good match out of 100 does not imply the other 99 are either good or bad. This only ensures that the method can find at least one good match, and even the averages plotted for most TF families are in the margin of acceptance. Therefore, in the next section (‘ModCRE predicts well in the twilight zone’) we will analyze more deeply the capacity of prediction.

We studied more thoroughly the prediction of 177 TFs that have motifs in JASPAR (with any method) and in CisBP (obtained only with PBMs). These TFs have 295 motifs in CisBP and 213 in JASPAR, which is about 20% of the original data with representation for most TF families. Considering that some TFs have more than one motif in JASPAR and in CisBP, the total number of comparisons between motifs was 369. All families except for TCR/CxC had at least one TF for which one model produced a PWM like the experimental (in JASPAR and CisBP). Table 3 shows the predicted motifs of a selected set of TFs compared with the experimental ones from JASPAR and CisBP. Despite our approach having learned the parameters of the predictive model using data from CisBP, the flexibility introduced with the variety of structural models helped to achieve good predictions of JASPAR motifs. In Supplementary Table S4 is shown a summary of the predictions for these TFs. From Table S4, considering successful the prediction for a TF if more than 50% of the predicted PWMs are significatively like the experimental motif, we correctly predicted almost 48% TFs’ motifs compared with JASPAR and 57% with CisBP. Then, if we consider successful the prediction for a TF if at least one of the predicted PWMs is significatively similar to the experimental motif, we predict 82% motifs compared with JASPAR and 89% with CisBP. In the next section we will apply these ideas to develop a new algorithm, like a jury-vote approach, to improve the accuracy and help in the selection of a single PWM and the detection of a binding site. Two facets of this validation with JASPAR must be noted: (i) We test the sequence of a TF blindly; therefore, although we have avoided TFs with more than one motif in JASPAR, the structural model can be produced by partial structures combining more than one domain (e.g. for sequences of the C2H2-Zf family with several domains). Then, an issue for this test is that for some TFs a portion of their models might correspond to regions of the protein addressed to a different binding site than the motif under test and consequently the comparison will fail. In addition, some structures contain more than one binding pose and some correspond to incomplete binding sites caused by the crystal (e.g. for the homeodomain family, the crystal structures of 2HOT, 2HOS, 1HDD, 2HDD, 3HDD and 1DUO from PDB show two different poses of the homeodomain binding, one of them incomplete), yielding the same problem. We assumed these problems as failures of the method because the region of the protein cannot be selected before the comparison with the real motif is done (we could compare the predicted PWMs and cluster them; nevertheless, selecting one or another cluster would still be random). (ii) The approach searches in the database of structures of TF–DNA complexes to obtain the structural model(s) of a TF. Therefore, finding a sufficiently similar TF sequence automatically imposes a specific conformation and sequence of the DNA. We can further stress this point by simply searching on a database of TF sequences with known DNA binding sites or motifs. This is known as nearest-neighbor approach (41,61) (see methods). In our validation, we neglected the DNA sequence of the template, but we assumed the same conformation in the model as in the template. A relatively large number of TFs preserve a B-DNA conformation (where subtle differences are unnoticed by the coarse-grained potential of our approach), but for some others, such as the TATA-box, the conformation in the template may be determinant of the sequence of the binding site. Therefore, we consider more appropriate to validate ModCRE by comparison with the nearest-neighbor (see next section). Finally, if we plan to use our approach on novel complexes without previous knowledge of the structure of the TF–DNA interaction, then the role of the DNA conformation must be considered, as this affects the quality of the prediction.

**Table 3.** Logos of experimental PWMs (from JASPAR and CisBP) and of their best predictions

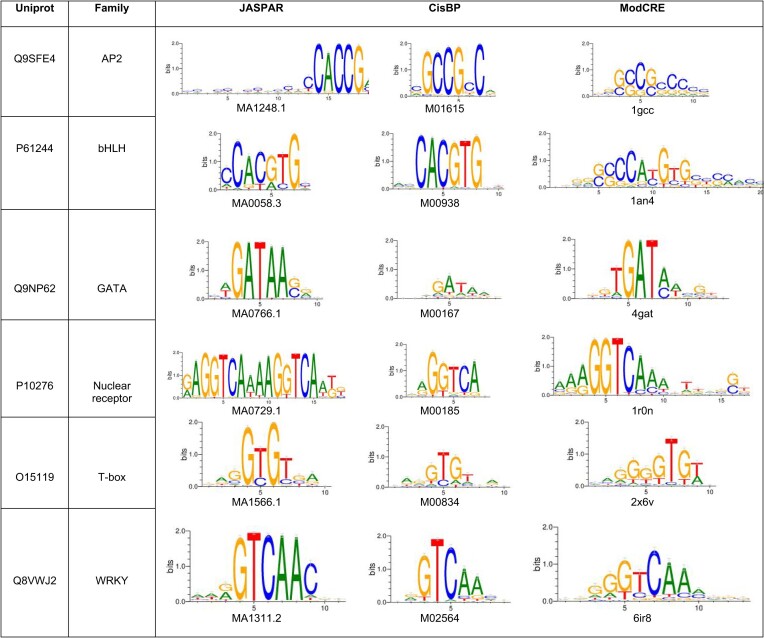

The table shows the logos of only some TFs for which the PWMs were also obtained by PBMs in CisBP. The TF is identified by the UniProt code. The code of the PWMs corresponding to the logos in JASPAR and CisBP are shown at the bottom. For the logos of the predicted PWMs the PDB code of the protein used as template is shown at the bottom. The logos of TFs from other families is shown in Supplementary Table S3.

### ModCRE predicts well in the twilight-zone

**Figure 3.**
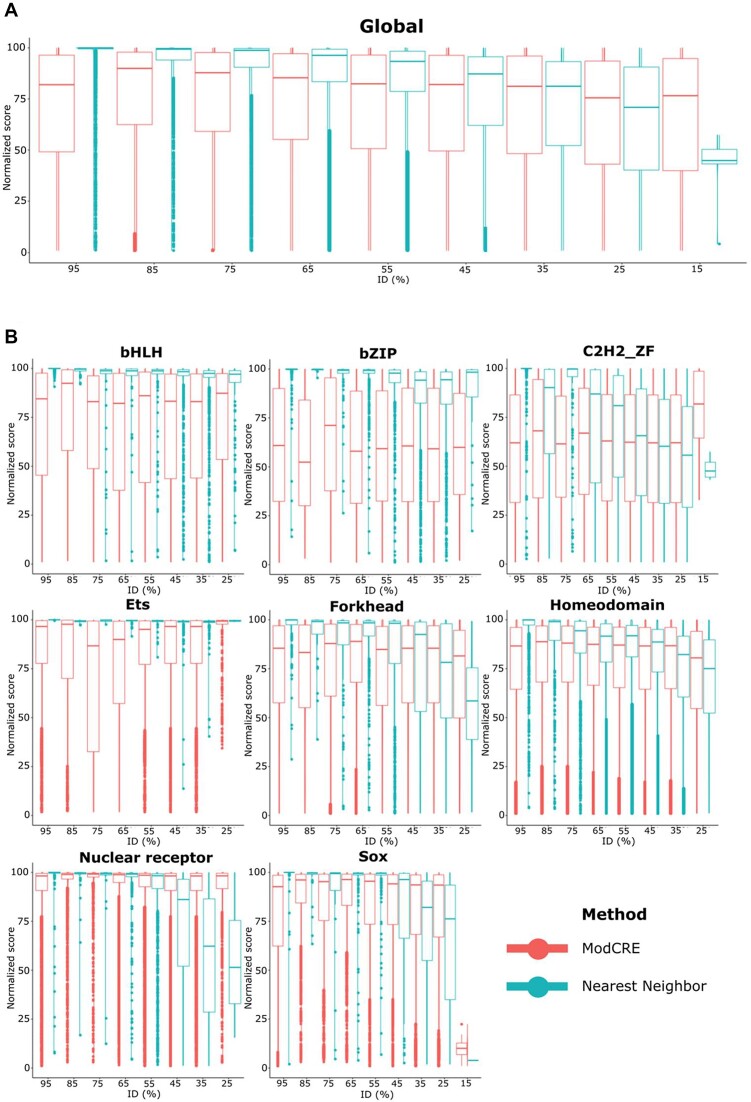
Distribution of the normalized ranking score of motif predictions with the nearest-neighbor approach and the structure-based approach (ModCRE). Results with the nearest-neighbor approach are shown in blue and results with the structure-based approach in red. The top distribution (**A**), entitled Global, corresponds to the distributions for all TFs. The distributions of normalized scores of some families of TFs are also plotted in (**B**) (the title of each plot indicates the name of the family). Plots of distributions of normalized scores for the rest of families can be downloaded from ModCRE website (http://sbi.upf.edu/modcre/##faq).

We simultaneously compared and validated the prediction of the PWM with the classical approach based on sequence homology. This approach is also known as prediction by nearest-neighbor (41,61). The nearest neighbor approach consists on using the experimental PWM of the closest homolog of a TF (41,61,62). The accuracy of such prediction depends on the degree of similarity between TFs: hypothetically, close homologs should have similar DNA binding domains and in consequence their PWMs should be similar too. As testing dataset, we used the TFs of CisBP that had been studied by PBMs. First, we compared their sequences using MMseq2 (42) (see Methods). Then, for each target sequence we grouped the other TFs of the dataset by sequence similarity with the target. Each group contains sequences that align with the target between a minimum and a maximum percentage of identical residues. The groups range between 15% and 95% binned in intervals of 10% (e.g. the group at 95% contains all closest homologs of a target TF which alignment produces a percentage of identical residues between 90% and 100%). The analysis of the prediction was performed by grouping TFs by families and the results by bins of the same interval of sequence similarity. Notice that it was not always possible to find relatives in all bins for all TFs. The total number of TFs in each bin and family varies, as well as the total number of predictions (homologs with a known PWM). Table S5 shows the number of TFs for which the nearest-neighbor approach can be applied and the total of predictions for each bin and family. For ModCRE, given a family and a bin, we used the same TFs that were tested in the nearest neighbor approach, for which we modelled 100 conformations (yielding 100 motifs). Then, instead of using the P-value as a criterion to test the predictions, we tested the success by ranking (see methods). The results with the predictions for all TFs are produced by the accumulation of results for all families in each bin (see Figure 3). The total number of TFs, motifs and predictions in each bin are shown in Supplementary Table S5.

The comparison between the nearest-neighbor and ModCRE clearly shows that the latter outperforms the nearest-neighbor approach at low sequence identity. For example, ModCRE predictions in the twilight zone, around 15–25% sequence identity, outperformed the nearest-neighbor approach for TF families such as C2H2 ZF, ETS and Homeodomains. For some families ModCRE outperformed the nearest neighbor approach at bins around 50% of sequence identity (i.e. families such as Forkhead, Nuclear Receptor and SOX). Nevertheless, for some families such as bHLH and bZIP, the nearest-neighbor method was still the best approach to predict their binding preferences, because these were preserved by distant and remote homologs. This was also reflected in the poor increase of diversity produced by new amino-acid and nucleotide contacts incorporated from PBMs experiments in the statistical potentials, showing that bHLH and bZIP families have a limited landscape of protein–DNA contacts that was easily covered by evolution. Interestingly, Lambert et al. (36) had already predicted by similarity regression the similarity in DNA sequence specificity between two TFs (specially for members of another family, the Homeodomain family), showing the relevance of the amino-acids in contact with the DNA, and helping to unveil the preservation of the binding motif between remote homologs. Lambert *et al.* (36) also noticed many highly or ambiguously similar homolog TFs in the bHLH and bZIP families, appearing rigid in their DNA-binding motifs and suggesting that TFs of these families had diversified through changes in heterodimerization partners. Consequently, we note that an acceptable error of the rank must be considered for each TF family specifically, because many TFs have PWMs like the motifs of other TFs in the same family. Hence, depending on the number of neighbors with similar PWM and how such similarity is defined, the rank can be confused with those of other very similar motifs. Supplementary Table S6 show the acceptable errors of the rank for several families of TFs. Acceptable errors in Table S6 are shown as a function of the *P*-value, *Q*-value and *E*-value to qualify if two PWMs are significantly similar. This helps us to determine the quality of predictions by nearest-neighbor and ModCRE approaches. Therefore, the evaluation by ranking permits us to compare the results of different TF families with independence of the size and variance of the PWMs of their TFs, while the margins of errors enable us to qualify the quality of the comparison with the predictions.

As mentioned above, having a successful prediction out of 100 models is useless when we don’t know the real PWM of the target. However, if a relevant number of models points to the same PWM (e.g. >50%), we could take this as the final prediction (e.g. by a majority vote selection). This suggests an additional criterion to predict the motif of a TF that can also be applied to the nearest-neighbor approach. We propose as solution (i.e. the predicted motif) the most often selected motif among the best rankings. For the nearest-neighbor approach, instead of selecting the PWM of the closest homolog, we consider all the motifs of TFs with sufficiently similar sequence to the target. Thus, a collection of motifs is used as in the structure-based approach. We name the approach ‘rank-enrichment prediction’ (see Methods). We must note that now a single solution is proposed for each TF in both approaches, nearest-neighbor and structure-based with ModCRE, and a single ranking value is obtained for the experimental motif of the target. Supplementary Figure S4 shows the distribution of the normalized rank-enrichment of the TF targets using ModCRE and the nearest-neighbor. The rank-enrichment prediction approach increases the accuracy of the predictions of both methods. ModCRE often achieves better coverage than nearest-neighbor at low percentages of similarity, while preserving most ranking scores at the top (around 98%). Supplementary Table S6 shows the acceptable errors in the normalized rank score as function of the *P*-value, *E*-value and *Q*-value for all families (e.g. when using all families, the average number of similar motifs with Q-value < 10^−3^ is around 60, and this implies a rank of about 98%, which is also in the same rank and with about the same number of similar motifs with *P*-value < 10^−7^). The acceptable errors for families such as Nuclear Receptors, C2H2-Zf, GATA or SOX are smaller than for Homeodomains. Interestingly, the enriched ranks of ModCRE predictions for TFs of Nuclear receptors and SOX families are high and the coverage is improved with respect to the nearest-neighbor approach.

### Comparison with other approaches (rCLAMPS)

A similar structural-learning approach, named rCLAMPS, was recently developed by Singh and cow. and applied on Homeodomain and C2H2-Zf families (43). They predicted the PWMs by mapping experimentally known PWMs in the contacts of the interface of a TF–DNA structure, which in principle (but not suggested) it could be applied to any other TF by providing the structure of the complex with DNA. We compared the capacity of ModCRE and rCLAMPS to predict the PWMs of TFs of the Homeodomain and C2H2-Zf families stored in JASPAR (see methods and supplementary Appendix for the implementation).

**Figure 4.**
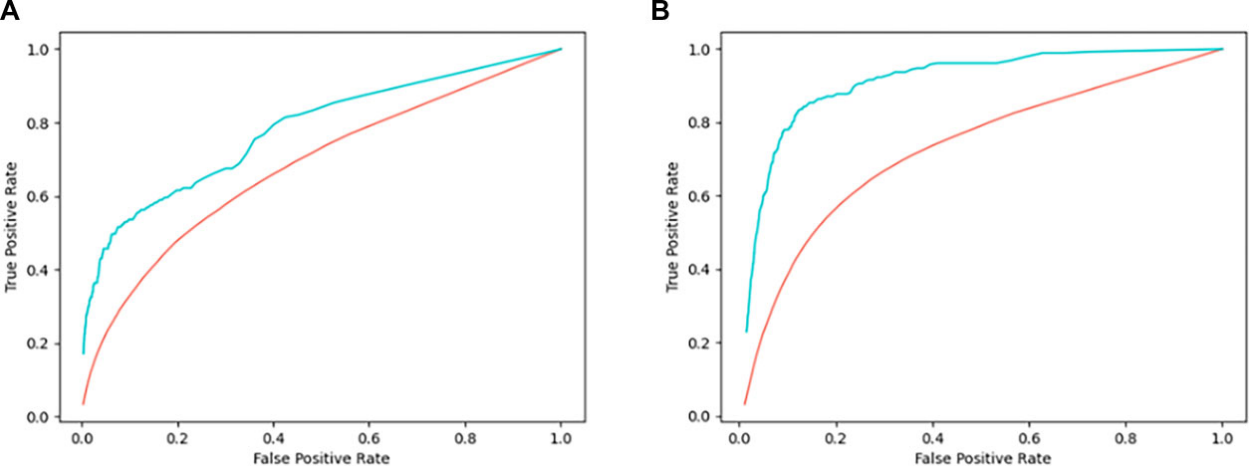
ROC curves for the prediction of PWMs with ModCRE and rCLAMPS. ROC curves are shown in red (ModCRE) and blue (rCLAMPS) continuous line. (**A**) ROC curve predicted for TFs of the C2H2 Zf family. (**B**) ROC curve predicted for TFs of the homeodomain family.

Figure 4 shows the ROC curves of both methods. The comparison highlights the good accuracy of rCLAMPS with respect to ModCRE. It's worth noticing that ModCRE was also prepared for the analysis and structural modeling of the bindings. Additionally, ModCRE showed significant accuracy to predict the PWMs of TFs when there weren’t close relatives available to apply the nearest-neighbor approach. Instead, the classical nearest-neighbor approach was highly accurate (and a better option) when the PWMs of close relatives were available. Here, rCLAMPS showed a better performance than ModCRE in global (i.e. both for TF with homologs that do or do not have a known PWM). Still, ModCRE had a good potential of prediction: the AUROC of the prediction of ModCRE for TFs of the Homeodomain family was 0.73 compared to 0.91 with rCLAMPS; and the AUROC with ModCRE for TFs of the C2H2-Zf family was 0.68, while with the predictions of rCLAMPS it was 0.78. Finally, ModCRE can be applied to all other TF families (predicting the binding sites with significant accuracy for some, such as the Nuclear Receptor, SOX or the bHLH families), and it can predict the binding site of any TF if the structure of the protein–DNA complex is provided.

### Application to predict bindings validated by ChIP-exo

In practice, unless the binding preferences of a TF have been experimentally tested, there is no access to the real motif(s) of a TF and consequently the rank-enrichment approach cannot be applied directly. However, we used the rank-enrichment approach to demonstrate that the best prediction is produced by a majority of similar motifs out of a collection of predicted PWMs. Therefore, a sensible approach to predict the binding site of a TF in a DNA sequence is: (i) to predict a set of PWMs, either by nearest-neighbor or with the structure-based approach; the latter if the homologs of the target are not sufficiently similar and the former if the percentage of sequence identity with the homolog is higher than 50%; (ii) scan the DNA sequence with all predicted motifs using FIMO (30) and (iii) select the fragment matched by the majority of motifs with a significant score.

**Figure 5.**
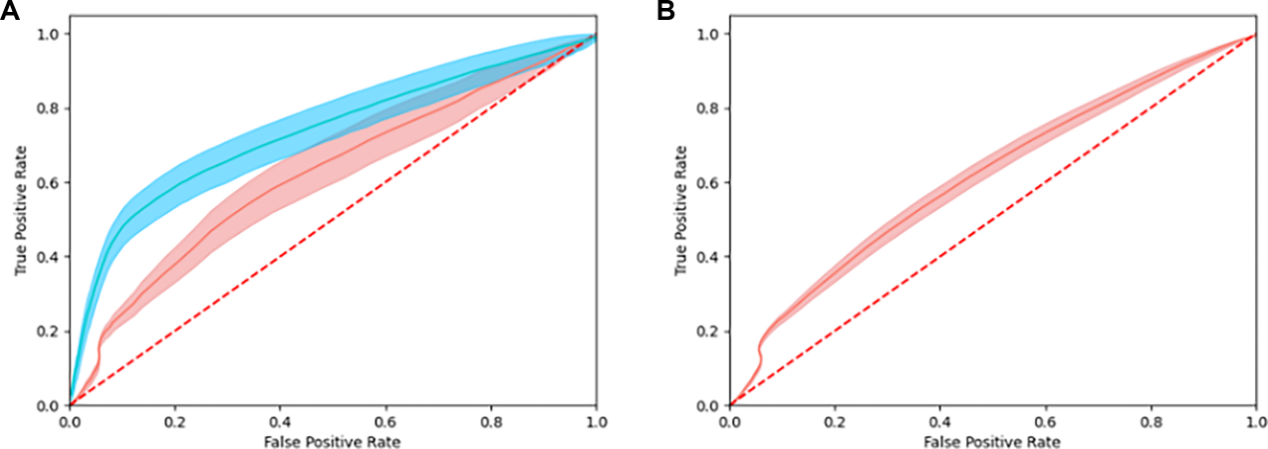
Average of ROC curves for the prediction of binding sites using ModCRE models and JASPAR motifs. (**A**) Averaged ROC curves using TFs with known motifs in JASPAR. Curves of the mean value are shown in red (ModCRE) and blue (JASPAR) continuous lines, and the standard errors are shown in their respective colors. (**B**) Averaged ROC curve and their standard error using TFs without known motifs in JASPAR. A discontinuous red line shows the ROC curve of a hypothetical random prediction. All TFs belong to the C2H2-Zinc finger family.

To ensure that ModCRE would be able to predict the transcription factor binding sites along a given sequence of DNA, we validated its prediction on experimentally known examples. We used ChIP-exo determined binding sites for Human transcription factors retrieved from the Gene Transcription Regulation Database (45) (see Methods). Figure 5 shows the average of ROC curves calculated with the prediction of the binding along a DNA fragment of around 400 bp. We selected only ChIP-exo experiments with a well-defined single-binding position (see methods and supplementary Appendix for details). The list of experiments and the UniProt IDs of the TFs used for this analysis is shown in Supplementary Table S7. Figure 5 shows the average of ROC curves characterizing the prediction for the selected experiments. Unfortunately, the set of experiments with sufficient accuracy for the benchmark contained studies only for TFs of the C2H2-Zf family. Despite this limitation, we could compare the accuracy of the prediction using the motifs from JASPAR and those predicted with ModCRE. Furthermore, we were able to test our hypothesis, using an ensemble of TF–DNA modelled conformations to generate several models and predicting the binding site with ModCRE by means of the ‘majority-vote’ rule (i.e. implementing a prediction that considers the accumulation of PWMs with a significant alignment around the potential binding site). Interestingly, the effect of the ‘majority-vote’ rule is shown in Figure 5A at the low FP ratio: First, using FIMO and the motifs from JASPAR with stringent thresholds (low *P*-values) the rate of TPs increases, while with ModCRE this is not possible (i.e. few modelled PWMs align, and many align in the wrong binding). Then, for significant but not too low P-values, the predictions with JASPAR motifs preserve the curvature of the rate of False and True positives, while the prediction with ModCRE has a subtle and relevant steep of accuracy produced by the effect of the accumulation of PWMs pointing to the correct binding site, without increasing the FP rate. This result can be improved either by using a larger number of models and/or by improving the accuracy of the predicted PWMs (e.g. combining ModCRE with approaches such as the nearest neighbor). Interestingly, the ROC curves with ModCRE, although not as accurate as with JASPAR motifs, have sufficient quality, with an AUROC of 0,67 while with JASPAR, assuming this is the maximum we can achieve experimentally, is 0,78 (which is only a difference of around 0.1 and it must be noted that the results using JASPAR motifs are based on experimental data).

Then, we evaluated the performance of ModCREs binding site prediction regardless of whether motifs were present in JASPAR or not. This is an excellent example of the applicability of our approach, as we increased the workable dataset from 84 experiments to 315. Due to the restriction on the significance of the experiment only the C2H2-Zf family was studied. Resulting ROC curves were averaged as above (see Figure 5B) and interestingly the AUROC was preserved (around 0.66, here we remind again that JASPAR motifs are not applicable, as there is no experimental data to do the prediction).

### Characterizing/identifying the binding sites of TFs

To analyze the binding sites of a DNA sequence, we have developed ModCRE as a web server that predicts the PWM of a TF based on its structure or by modelling several conformations with its sequence. Then, the DNA sequence can be scanned with the PWMs and either the accumulation of matches can be profiled (i.e. using a score for the prediction of binding), or a selection of matches can be collected to build the structure of a cooperative binding (i.e. considering the formation of a potential complex of transcription). The webserver permits the substitution of the predicted PWM by another (in MEME format). This is more convenient if we know experimentally the PWM or if we know the PWM of a close homolog of the target (as it had been shown in the previous sections). To facilitate the scanning of a large DNA sequence and the construction of a structural model of cooperative binding, we have included the PWMs of three datasets associated with TF structures. Two datasets are defined with the experimental motifs from JASPAR and CisBP. In each of these sets a motif is associated with the sequence of a TF. Therefore, we have modelled the potential conformations of each one of these TFs and selected the model with the PWM most similar to the experimental motif. Then, a target DNA sequence is scanned with FIMO using the motifs of the corresponding database and the associated models can be selected in ModCRE web server. Similarly, the third database is obtained using the structures of TFs complexed with a DNA double strand: we use BLAST (26) and HMMER (63) to obtain the sequences of potential homologs in UniProt and TrEMBL (34) that align (without gaps in the binding interface) with the sequences of these TFs. A motif is predicted for any of these sequences using the alignment and the template structure. Consequently, we can scan the DNA with any of the sequences of specific species (see further details in Supplementary Appendix). Additionally, specific TF sequences can be uploaded to predict their motifs and scan the DNA.

### Integration of transcription factors and co-factors in a regulatory complex

To complete the modelling, co-factors can be included in the network of interactions when the species selected are human or mouse. Interactions between TFs and transcription co-factors (TcoFs) are retrieved from the TcoF-DB database (64). After selecting a set of protein–protein and protein–DNA interactions, these can be modeled using a homology modeling pipeline (49,57). Then, ModCRE models the structure of DNA in a specific conformation (B conformation by default), and for very long DNA sequences the server splits the sequence in fragments of 250 bp (with an overlap of 50bp to be able to assemble them later). Models with clashes between proteins are removed and only acceptable combinations of each fragment are selected to construct a model. Next, the structures are optimized by several steps of conjugate gradient and short annealing dynamic simulations with MODELLER. Finally, distance restraints are extracted from the models of protein–protein interactions and TF–DNA interactions, and we use the package IMP (65) to integrate them in a model of DNA with all TFs and TcoFs (see more details in Supplementary Appendix). We are not aware of any other web-service method to automate the structural modeling of these complexes and very few experimentally known complexes to benchmark. The most similar approach was recently described if RoseTTAFold2NA (66).

### Example 1: the interferon-beta (IFN-β) enhanceosome

**Figure 6.**
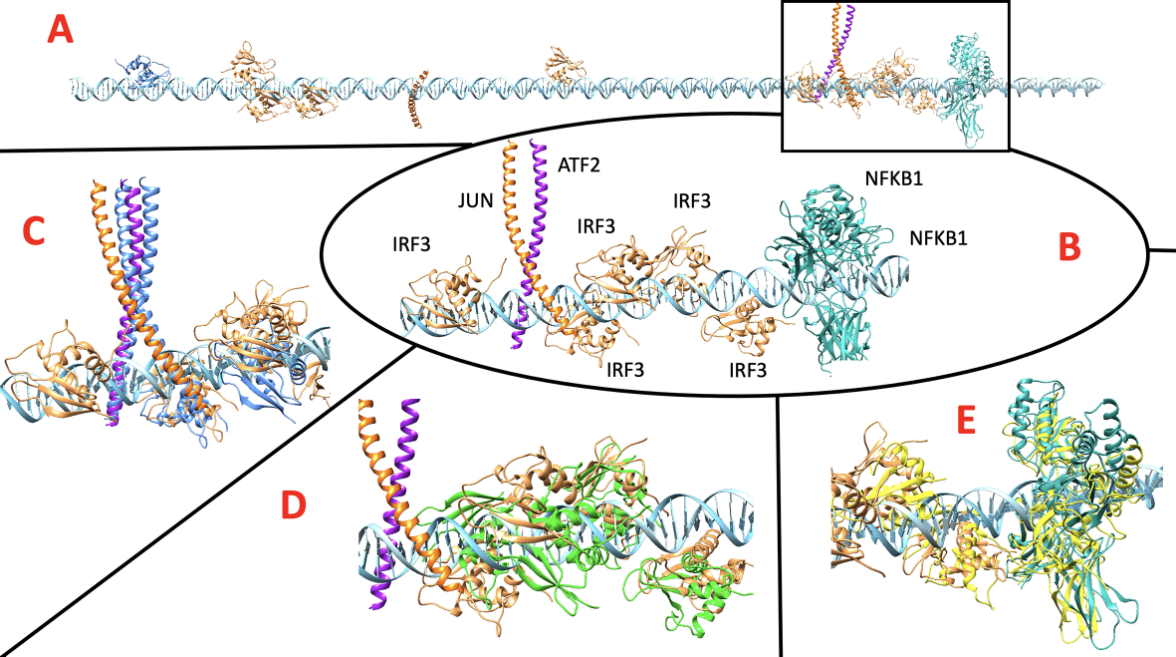
Model of the IFN-β enhanceosome complex. The structure of the complex formed by interactions between proteins and DNA is automatically built with the selected TFs and their binding sites in the enhancer sequence of IFN-β (Supplementary Figure S5). (**A**) Model of one of the complex structures obtained with the largest number of TFs while avoiding clashes between them. Due to the large time of computation, not all combinations of distinct conformations, produced by different templates, are tested. The structures of ATF-2 (purple), c-JUN (orange), IRF-3 (yeast) and NFKβ-1 (light blue) are shown on their binding with DNA (cyan), highlighting in a squared framework the region corresponding to the model of the enhanceosome proposed by Panne (21,67). (**B**) Detail of the automated model obtained with ModCRE (IRF-3 is indicated as IRF3, ATF-2 as ATF2, c-JUN as JUN and NFKβ-1 subunit p105 as NFKB1): i) IRF-7 is missing; ii) NFKβ-1 forms a homodimer of two p105 subunits instead of the expected heterodimer with subunit p65 (RelA); and iii) an extra IRF-3 is bound at 5′ of the ATF-2/c-Jun binding. (**C**) Detail of the superimposition of the model of ModCRE with the crystal structure of ATF-2/c-Jun and IRF-3 bound to the interferon-beta enhancer (code 1T2K (79) from PDB, shown in blue). (**D**) Detail of the superimposition of the model with the crystal structure of IRF-3 bound to the PRDIII-I regulatory element of the human IFN-β enhancer (code 2PI0 (81) of PDB, shown in green). (**E**) Detail of the superimposition of the automated model with the crystal structure of NFKβ-1 (subunits p105 and p65), IRF-7, and IRF-3 bound to the IFN-β enhancer (code 2O61 (67) of PDB, shown in yellow).

We have used the server to automate the modelling of the interferon-beta (IFN-β) enhanceosome, an ensemble of TFs and Cis-regulatory elements that cooperate in the enhancer of the IFN-β gene (21,67). The TFs binding at the IFN-β enhanceosome are ATF-2, c-Jun, IRF-3, IRF-7, and NFKβ-1 (subunits p105 and p65). We have used a sequence of 250bp containing the region of the enhanceosome and the database of human TFs, using their predicted PWMs based on their modelled structures, to predict and model the co-operative complex. These PWMs are used to scan the DNA sequence with FIMO. In Supplementary Figure S5 we select the bindings of the specific TFs (i.e. ATF-2, c-Jun, IRF-3, IRF-7 and NFKβ-1) that are significant (*P*-values < 5.0e^−4^). Hence, we are able to recreate a structural model similar to the model provided by Panne (21,67) based on experimental data. On the binding sites predicted for NFKβ-1 (subunits p105 and p65) the automated approach produces a homodimer of NFKβ-1 with two subunits p105 instead of a heterodimer with RelA (subunit p65). Not all the binding regions of IRF-3 are detected exactly, but some other regions are predicted instead. Besides, the IRF-7 binding site from Panne's model is occupied by IRF-3. The analysis highlights the accumulation of TFs in a short section of the DNA and brings a potential explanation for the formation of the transcription complex by gathering TFs (Figure 6).

### Example 2: TF–DNA interactions on top of the nucleosome

**Figure 7.**
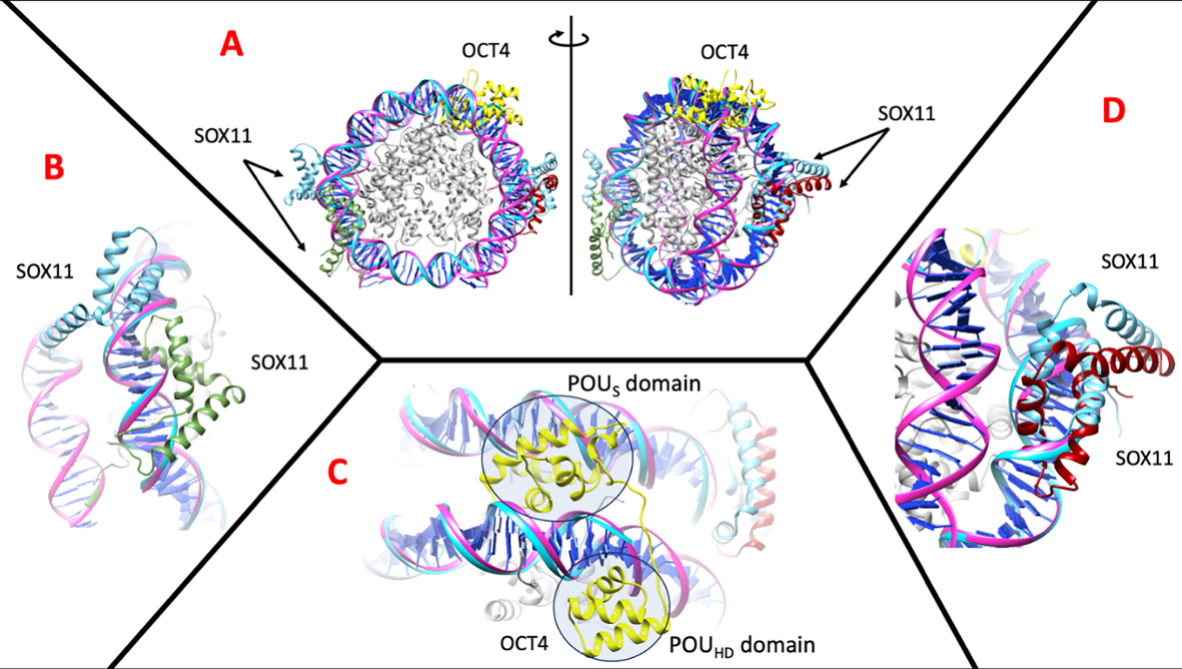
Model of the nucleosome complex with SOX11/SOX2 and OCT4. (**A**) Front (left) and lateral (right) view of the complex obtained automatically with ModCRE superposed with the experimental structure (code 6T7C in PDB). Both model and experimental structures have only SOX11 bound (indicated as SOX11) to the nucleosome. The conformations modelled with ModCRE are shown in green (binding between 54 and 64 bp) and dark red (binding in the interval 86–96 bp); the conformations of SOX11 from the experimental structure are shown in blue (binding in the interval of 50–60 bp and 86–96 bp, respectively); and the model of OCT4 (indicated as OCT4) is shown in yellow (binding along an interval of 23–35 bp) indicating encircled the POU Homeodomain (POU_HD_) and POU Specific (POU_S_) domains. (**B**) Detailed comparison of the binding of SOX11 between the automated model (green) and the experimental structure (blue). The predicted PWM fails to position neither SOX11 nor SOX2 around the 50 bp (the closest fragment is in the interval of 54–57 bp). (**C**) Detail of the modelled binding of OCT4 (in yellow). Two domains of OCT4 produce the binding in a large interval of the DNA sequence (between 23 and 35 bp), where the domain that binds in 3′ (around 28–35 bp) has also ‘non-binding’ contacts (with many clashes) with the DNA fragment around 98–112 bp. This suggests a potential weakening of the nucleosome complex conformation that could lead to unfasten it. (**D**) Similar binding of SOX11 around 86–96 bp of the model (dark red) and experimental (blue) structures. The DNA sequence used in the model is DNA1 from the study of Dodonova *et al.* (22): *ATCTACACGACGCTCTTCCGATCTAATTTATGTTTGTTAGCGTTATACTATTCTAATTCTTTGTTTCGGTGGTATTGTTTATTTTGTTCCTTTGTGCGTTCAGCTTAATGCCTAACGACACTCGGAGATCGGAAGAGCACACGTGAT*.

An interesting case of co-operation between TFs are the ‘pioneer factors’ (the first to engage target sites in chromatin culminating in transcription by displacing nucleosomes (68)), or TFs that can bind on top of the nucleosome complex (as detected by NCAP–SELEX (69)). A structural view of this complex has shed light on the characteristics of the TF–DNA interaction and its effect upon the conformation of the nucleosome (22). The server also has the possibility to produce a bent conformation such as the nucleosome that includes histones to form the complex (IMP is not applied because the structure of DNA is already defined). We have used the automated modelling of a nucleosome in complex with SOX2, SOX11 and OCT4 from the study of Dodonova *et al.* (22) to analyze these ‘pioneer factors’. Interestingly, we found two very significant binding regions of SOX2 and SOX11 (*P*-value < 1.0e^−4^, around 58 and 85 bp positions) with the PWM predicted by ModCRE, but they were hampered by histones. We also found three sites with less significance (*P*-value < 1.0e^−3^) but accessible to SOX11/SOX2 and OCT4 (SOX11 and SOX2 share the same binding site preferences). However, SOX2 is missing in the predicted complex and the models of SOX11 and OCT4 clash with the DNA, implying the need of a posterior distortion to produce the complex (Figure 7). For OCT4 the model recreates the binding with both domains, but we must notice that with only one domain the binding is possible without producing clashes in the nucleosome second turn of DNA (in 98–112 bp). To explore the binding of OCT4, we modeled the interaction among the pioneering TFs OCT4, SOX2 and KLF4 in the transcription of the Lin28B gene, known for its high expression in various tissues and cancer cell lines. The promoter region of Lin28B adopts a nucleosome conformation (PDB codes 7U0I and 7U0G) (70). Our modeling revealed two OCT4 domains, POU_HD_ and POU_S_, binding to DNA fragments proximal to the position of OCT4 in 7U0G (around nucleotides 140–155 in chain I). Conversely, in the position of OCT4 in 7U0I (around nucleotides 145–155 in chain I), our prediction suggests binding by SOX2. Additionally, we predicted and modeled another POU_HD_ domain in position 65–75 of 7U0G in chain I, closely resembling the experimental location of OCT4 in 7U0I (corresponding to nucleotide positions 75–85 of chain I). Two SOX2 domains were also modeled around nucleotides 130–140 and 50–60 of 7U0G in chain I, with an experimental POUHD domain of OCT4 located at 55–65 of 7U0G in chain I. Furthermore, an experimental full OCT4 (comprising POU_HD_ and POU_S_ domains) was identified around nucleotides 30–40 of 7U0I, chain I, and we predicted a POU_HD_ domain in the same region. Finally, near this region, in position 110–120 of 7U0I, chain I, our prediction modellates two zinc-finger domains of KLF4 (see Supplementary Figure S6 for detailed representation).

## Conclusion

Knowledge of TF-binding specificities is the foremost condition to understand gene regulation. Still, the binding preferences for many eukaryotic TFs are unknown or very complex *in vivo* (7,71). In this regard, computational tools can complement experimental methods. Several approaches have been taken in the recent years to computationally predict the binding of certain TF families such as the C2H2-Zf (72) or homeodomains (73). Other approaches have also considered the use of statistical and molecular-mechanics potentials (74,75). In this work we have developed a structure-based approach to predict specific binding motifs of TFs, to identify *cis-*regulatory elements and to automatically model the structure of the transcription complex entailing the regulation. Our approach has been implemented in a server for the scientific community named ModCRE. The main limitation of these approaches is that they can only be applied to TFs for which the structure of the interaction with DNA is known. The scarcity of these experimental structures also affects the number of templates to be used by homology modeling. As shown in Supplementary Figure S7, ModCRE can be applied to most TF families defined in UniProt and, in combination with JASPAR database, our approach can be applied to 88.7% of the TF sequences. Besides, thanks to the advent of AlphaFold2 (20,76) the structure of almost all TFs can be predicted, and remarkably the DNA binding motif, that often contains a large percentage of regular secondary structures, can be build. We have tested the percentage of TFs from the database AnimalTFDB 4.0 (77) for which each approach can be applied. We have analyzed a total of 274 633 TFs. We determined that the nearest neighbor approach could be effectively employed with sufficient accuracy if at least one homolog of the TF could be located in JASPAR, aligning with a minimum of 50% identical residues. This method was applicable to 71% of the TFs. Alternatively, if this criterion was not met, we considered utilizing homology modeling and ModCRE to predict the structure of the TF, provided that at least one homologous structure could be identified in ModCRE, aligning with at least 30% identical residues. This approach was applicable to 23% of the TFs. For the remaining TFs, where neither of these methods was viable, we proposed employing AlphaFold2 to construct a structural model of the TF–DNA interaction. Subsequently, ModCRE could be utilized to predict the PWM using the generated structure. This strategy was applicable to the remaining 6% of TFs. It's worth noting that these percentages varied across different TF families, as illustrated in Supplementary Figure S8. Furthermore, as observed in Supplementary Figure S2, the data from PBM experiments significantly enhances the accuracy of family-specific statistical potentials in predicting binding. This highlights the necessity of incorporating more of this experimental data to improve predictive accuracy.

**Figure 8.**
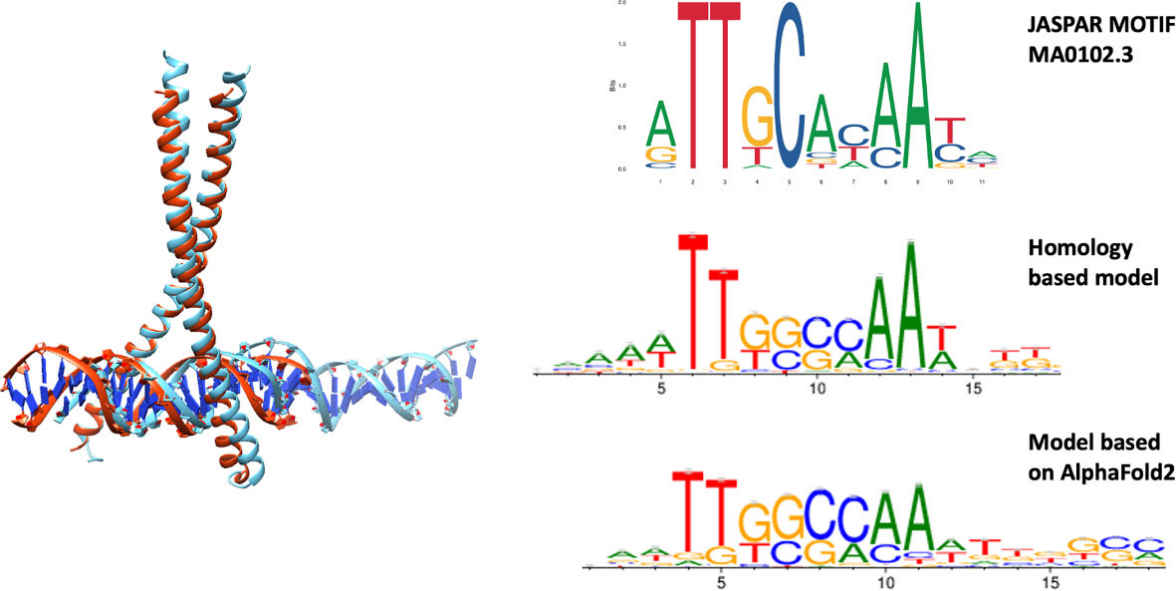
Prediction of PWMs of human C/EBPα. The ribbon plate structures on the left side show the superposition of a model of human C/EBPα (red) obtained with the crystal structure of rat (code 1NWQ in PDB) as template and a model based on the structure predicted with AlphaFold2 (blue). The right side shows a comparison of the motif logos of human C/EBPα: i) the top logo corresponds to motif MA0102.3 from JASPAR; ii) the middle logo is obtained with the prediction based on the homology model of human C/EBPα and iii) the bottom logo is obtained with the structure modelled upon the structure prediction of human C/EBPα obtained with AlphaFold2.

The structure of any TF–DNA complex can be modelled either by docking or by superposition with other members of the TF family. As a tailored example we have studied the human motif of the CCAAT/enhancer-binding protein alpha (*C/EBPα*.). The human protein has not been crystallized, but the DNA binding motif of rat is 100% identical and the structure of the dimer is available in PDB with code 1NWQ (78). We downloaded the AlphaFold structure of *C/EBPα*. from human (AF-P49715-F1 from UniProt) and selected only the DNA binding domain (α-helix residues 284–344). We superposed this domain on each chain of the structure of the heterodimer of ATF-2 and c-Jun (PDB code 1T2K (79)) to get the dimer complex of *C/EBPα*. with DNA. We used both structures to predict the PWM with ModCRE, one by submitting the sequence and getting the motif with 1NWQ as template, and the other by submitting the structure. This example shows almost identical motifs (see Figure 8). In this line, the latest versión of RoseTTAFold, RoseTTAFoldNA (66), has incorporated the *ab initio* modelling of the structure of protein–DNA interactions that could be used straightforward in ModCRE to predict the PWM and scan one or more DNA sequences.

**Figure 9.**
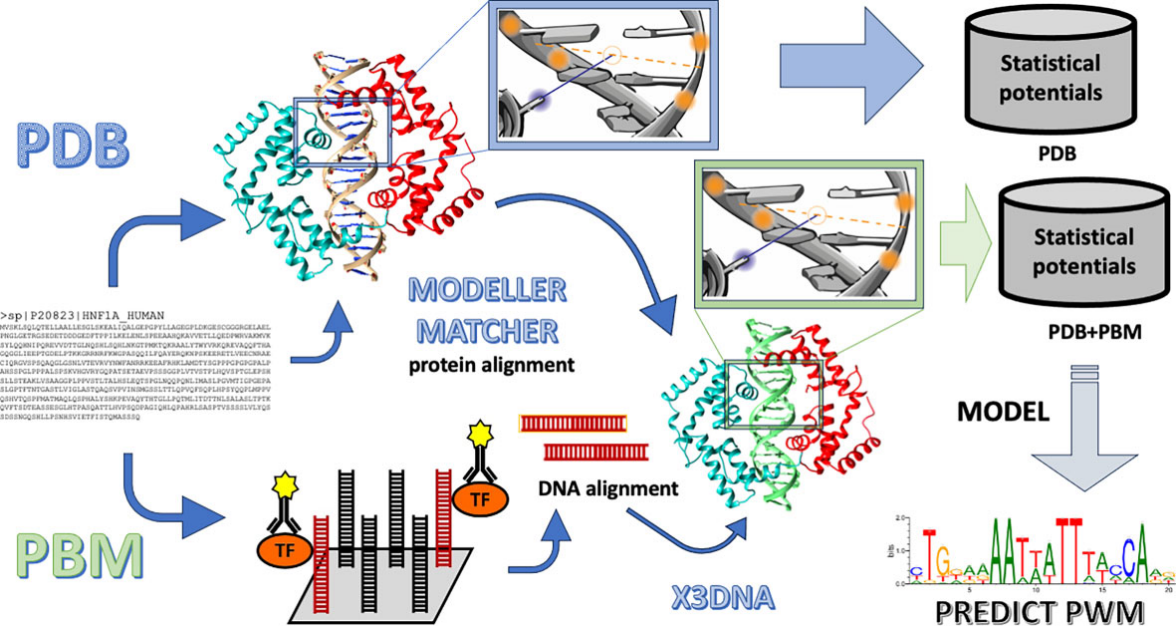
Schema of ModCRE approach. The approach is applied to all TF sequences in CisBP (the sequence of human HN1FA is shown as example). We use the known structures of TF–DNA complexes in PDB to collect the contacts between amino-acids and dinucleotides. We model additional contacts using experimental interactions of each TF collected from PBMs and structural models of its close homologs. The contacts are used to generate various statistical potentials applying several conditions. The best parameters for each specific TF family are selected to construct a prediction model that scores the binding of any TF–DNA complex structure. A known or modelled structure of TF–DNA complex is tested with all possible DNA binding sequences heuristically, which are then ranked and aligned, yielding a multiple-sequence alignment, to predict the PWM.

By incorporating the structural variability and flexibility of a TF we have designed an improvement of the prediction of its binding-sites based on the largest preference of motifs, each motif generated with one conformation. Thus, by scanning with several theoretical motifs of a TF, the majority of regions detected and predicted to bind will hit around the right location of the binding site. Using a collection of motifs derived from different models of a TF is in consonance with the idea that TFs can interact with the DNA adopting different conformations (7). Not only the dynamics of the protein but also the flexibility of the conformation of the DNA plays a relevant role in the identification of the binding site. Molecular dynamics of such complexes have recently been used to predict the binding affinity of TFs and to predict its corresponding PWMs (80). We used homology modelling in the same line in our approach. Homology modelling is very convenient when several templates are available because it generates a collection of models of a TF without requiring large computational resources. Still, the time of computation to calculate the PWM in the server is between 30 min and 5 h (depending on the size of the interface). Similarly, AlphaFold can be applied to obtain several conformations. In agreement with this, ModCRE’s modeling pipeline is a valuable resource to study the conformations of large regulatory complexes. Structural models of TF–DNA interactions provide fundamental information to understand TF function and behavior. Our pipeline models complexes of TF–DNA interactions involving DNA bindings and protein–protein interactions between TFs and transcription co-factors. We hypothesize that the correct binding site is among the selection of sites where most conformations of TFs accumulate when considering the cooperation with other TFs and co-factors. A drawback for the server is that the final steps to model the macro-complex require a large time of computation. Nevertheless, this may be a promising strategy helping to overcome the number of false positives found when scanning a DNA sequence with a single PWM (7,71) or at least to narrow the predictions and simultaneously comprehend the cooperativity between transcription factors and co-factors.

## Supplementary Material

lqae068_Supplemental_Files

## Data Availability

Software: https://figshare.com/search?q=modcre. Server (additional data in FAQS): http://sbi.upf.edu/modcre.
