# Supplementary material for: Structure-based learning to predict and model protein–DNA interactions and transcription-factor co-operativity in cis-regulatory elements
Source: NAR Genom Bioinform. 2024 Jun 12;6(2):lqae068. doi: 10.1093/nargab/lqae068 (PMC11167492; doi:10.1093/nargab/lqae068)
Supplement: lqae068_Supplemental_Files [file lqae068_supplemental_files.zip › TableS3.pdf]

Uniprot	Family	JASPAR	CisBP	ModCRE
Q9SFE4	AP2	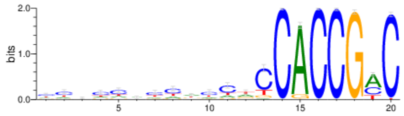 MA1248.1	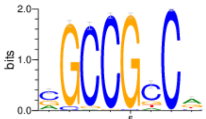 M01615	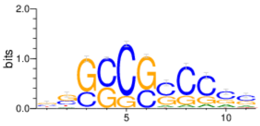 1gcc
P61244	bHLH	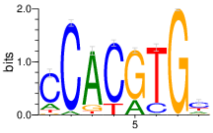 MA0058.3	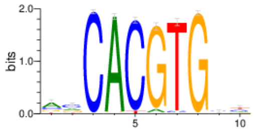 M00938	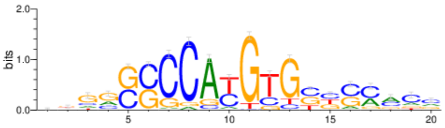 1an4
P17535	bZIP	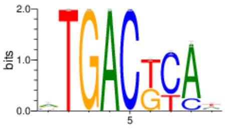 MA1143.1	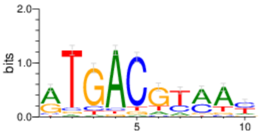 M01001	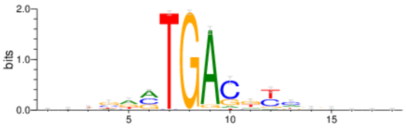 2h7h
P22561	C2H2-ZF	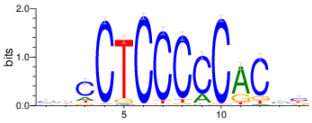 MA1627.1	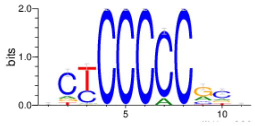 M00249	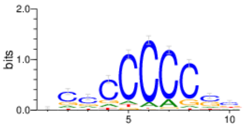 2jp9
Q8IXT2	DM	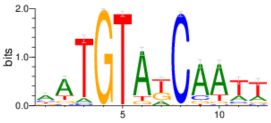 MA1479.1	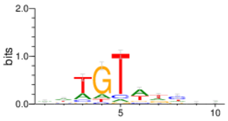 M01933	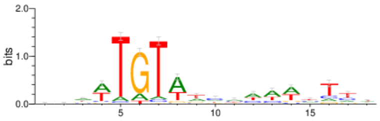 4yj0
P41161	Ets	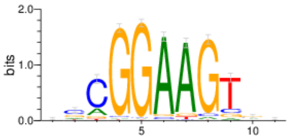 MA0765.2	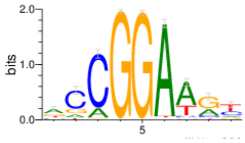 M01485	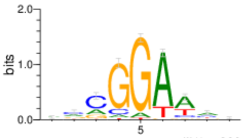 4uuv
Q9R1E0	Forkhead	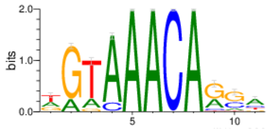 MA0480.1	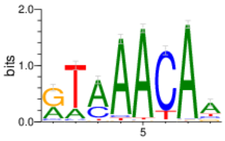 M00797	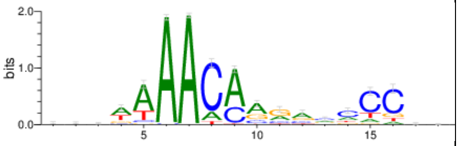 4lg0
Q9NP62	GATA	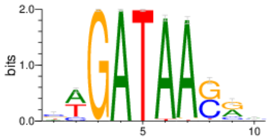 MA0766.1	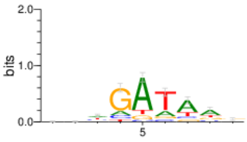 M00167	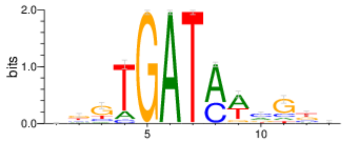 4gat
Q9NP62	GCM	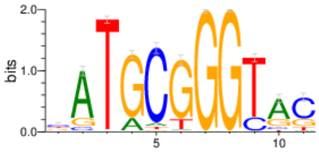 MA0646.1	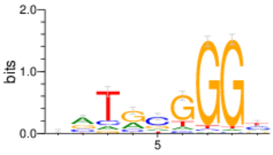 M02020	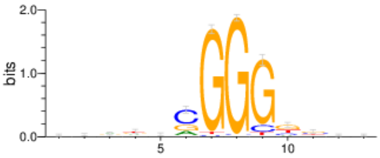 1odh

Uniprot	Family	JASPAR	CisBP	ModCRE
Q9UBX0	Homeo-domain	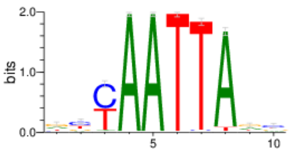 MA0894.1	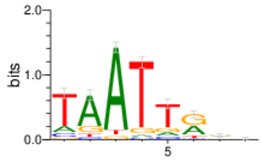 M00307	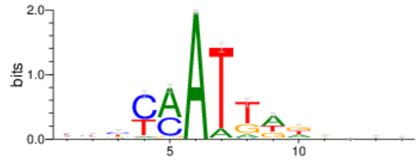 3lnq
O43316	Homeo-domain Paired box	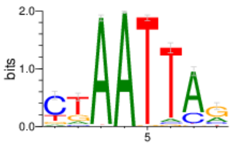 MA0068.2	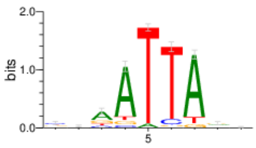 M00349	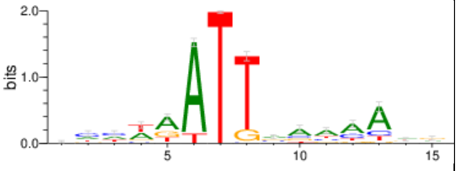 5no6
Q14863	Homeo-domain POU	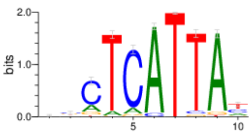 MA1549.1	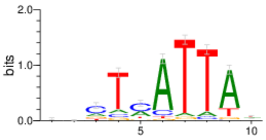 M00546	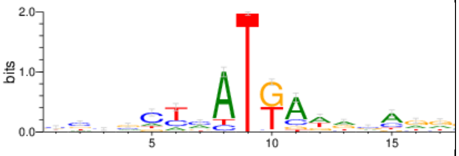 2r5y
Q03933	HSF	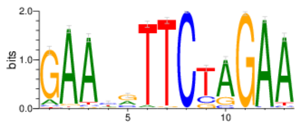 MA0770.1	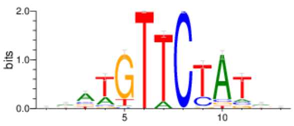 M02248	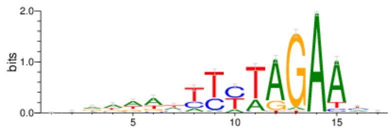 5d8l
Q92985	IRF	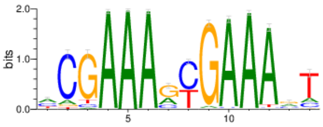 MA0772.1	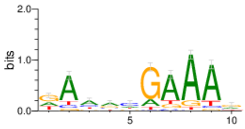 M02255	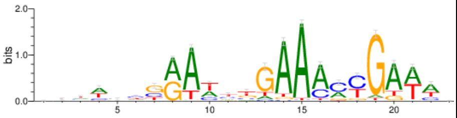 1t2k
P10243	Myb/SANT	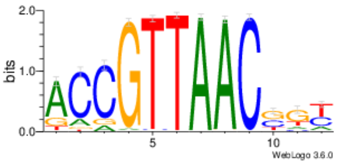 MA0776.1	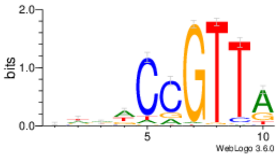 M02322	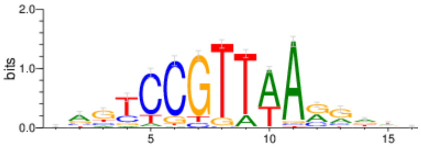 1h88
P10276	Nuclear receptor	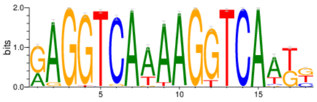 MA0729.1	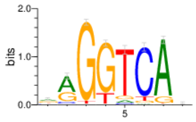 M00185	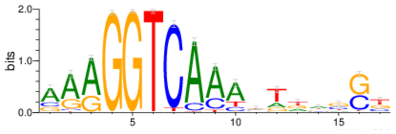 1r0n
P32114	Paired box	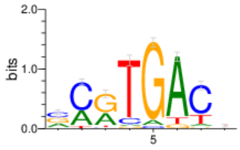 MA0067.1	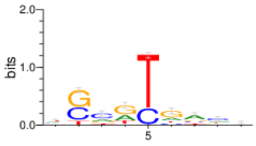 M01301	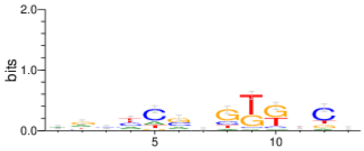 1pdn

Uniprot	Family	JASPAR	CisBP	ModCRE
G1MTN6	Rel	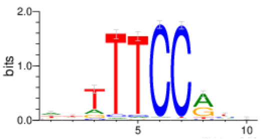 MA0624.1	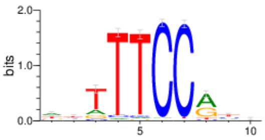 M02443	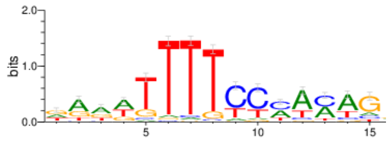 2o93
Q13950	Runt	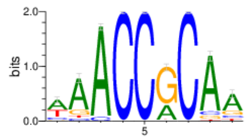 MA0511.2	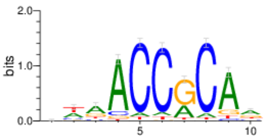 M01307	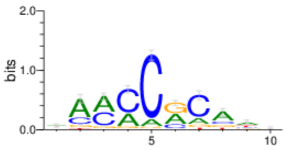 1h9d
P40645	Sox	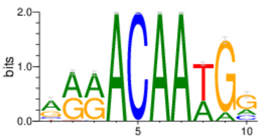 MA0515.1	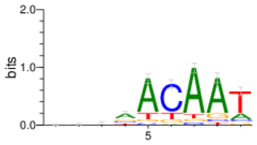 M00828	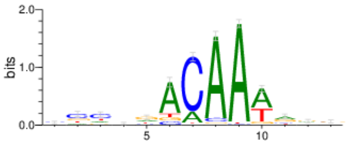 4s2q
O15119	T-box	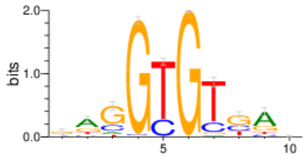 MA1566.1	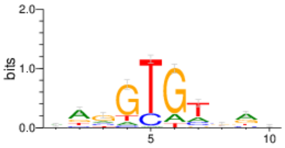 M00834	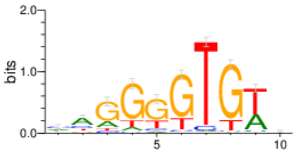 2x6v
Q95QD7	TCR/CxC	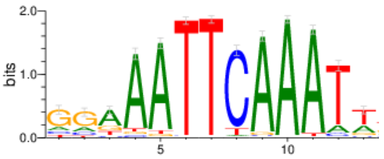 MA1450.1	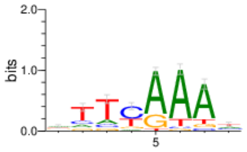 M02526	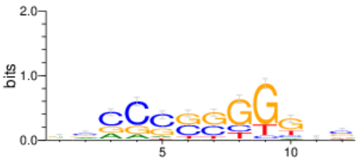 5fd3
Q8VWJ2	WRKY	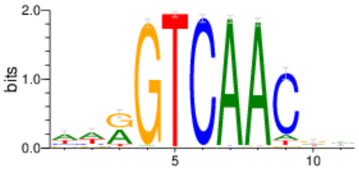 MA1311.2	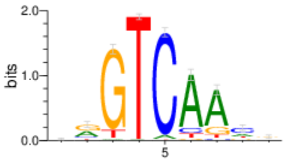 M02564	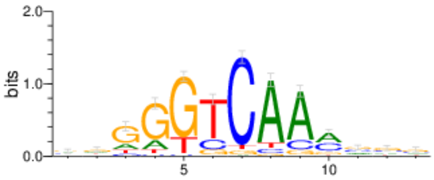 6ir8
